# Simple Generation of Suspensible Secondary Microplastic Reference Particles via Ultrasound Treatment

**DOI:** 10.3389/fchem.2020.00169

**Published:** 2020-03-18

**Authors:** Elisabeth von der Esch, Maria Lanzinger, Alexander J. Kohles, Christian Schwaferts, Jana Weisser, Thomas Hofmann, Karl Glas, Martin Elsner, Natalia P. Ivleva

**Affiliations:** ^1^Institute of Hydrochemistry, Chair of Analytical Chemistry and Water Chemistry, Technical University of Munich, Munich, Germany; ^2^Chair of Food Chemistry and Molecular Sensory Science, Technical University of Munich, Freising, Germany

**Keywords:** secondary microplastic, reference material, fragmentation, polystyrene (PS), polyethylene terephthalate (PET), polylactic acid (PLA), suspensible, Raman microspectroscopy

## Abstract

In the environment the weathering of plastic debris is one of the main sources of secondary microplastic (MP). It is distinct from primary MP, as it is not intentionally engineered, and presents a highly heterogeneous analyte composed of plastic fragments in the size range of 1 μm−1 mm. To detect secondary MP, methods must be developed with appropriate reference materials. These should share the characteristics of environmental MP which are a broad size range, multitude of shapes (fragments, spheres, films, fibers), suspensibility in water, and modified particle surfaces through aging (additional OH, C=O, and COOH). To produce such a material, we bring forward a rapid sonication-based fragmentation method for polystyrene (PS), polyethylene terephthalate (PET), and polylactic acid (PLA), which yields up to 10^5^/15 mL dispersible, high purity MP particles in aqueous media. To satisfy the claim of a reference material, the key properties—composition and size distribution to ensure the homogeneity of the samples, as well as shape, suspensibility, and aging —were analyzed in replicates (*N* = 3) to ensure a robust production procedure. The procedure yields fragments in the range of 100 nm−1 mm (<20 μm, 54.5 ± 11.3% of all particles). Fragments in the size range 10 μm−1 mm were quantitatively characterized via Raman microspectroscopy (particles = 500–1,000) and reflectance micro Fourier transform infrared analysis (particles = 10). Smaller particles 100 nm−20 μm were qualitatively characterized by scanning electron microcopy (SEM). The optical microscopy and SEM analysis showed that fragments are the predominant shape for all polymers, but fibers are also present. Furthermore, the suspensibility and sedimentation in pure MilliQ water was investigated using ultraviolet–visible spectroscopy and revealed that the produced fragments sediment according to their density and that the attachment to glass is avoided. Finally, a comparison of the infrared spectra from the fragments produced through sonication and naturally aged MP shows the addition of polar groups to the surface of the particles in the OH, C=O, and COOH region, making these particles suitable reference materials for secondary MP.

## Introduction

The characterization of microplastics (MP)—i.e., of small synthetic polymer particles—is a four-dimensional challenge, consisting of (I) the broad size distribution of particles (and fibers) from 1 μm to 1 mm, (II) the variety of polymer types and natural particles, (III) the state of aging, and (IV) the variety of forms (spheres, films, fragments, fibers) (Hartmann et al., 2019). All four dimensions should ideally be detected and quantified simultaneously in one measurement (Ivleva et al., 2017; Anger et al., 2018; Koelmans et al., 2019). The need to develop suitable analytical tools is accompanied by the need for effective methods to produce reference particles—reference materials that should be as similar as possible to the MP particles found in environmental and food samples.

Plastic undergoes aging processes in the environment that cause fragmentation into secondary MP (Brandon et al., 2016). In contrast to primary MP, secondary MP is not produced in a targeted manner but originates from aging and fragmentation of polymer materials and thus results in a heterogeneous mixture of particles. If Raman or infrared (IR) spectroscopy are used for analysis, the vibrational spectra of the particles may not match conventional databases since they may contain hydroxy, carbonyl, and carboxy groups in addition to the pure polymer spectrum, as an effect of environmental aging (Brandon et al., 2016; Lambert and Wagner, 2016; Cai et al., 2018). Furthermore, environmental MP is suspended in bodies of water, such as fresh (Eriksen et al., 2013; Imhof et al., 2013, 2016; Free et al., 2014; Dris et al., 2015; Eerkes-Medrano et al., 2015) and marine waters (Hidalgo-Ruz et al., 2012; Enders et al., 2015; Frere et al., 2017; Erni-Cassola et al., 2019). In the nanometer range the suspension of reference particles can be achieved by the addition of surfactants (Balakrishnan et al., 2019) or can result from the particle generation procedure, as shown by Magri et al. (2018) for their nano polyethylene terephthalate (PET) generated by laser ablation or by Pessoni et al. (2019) for their nano polystyrene (PS) particles from soap-free emulsion polymerization. These effects are much harder to achieve for MP reference particles (1 μm−1 mm). The current state of the art to generate particles in this size regime is cryo milling (Eitzen et al., 2018). These particles are not easily suspended and the difficulty arises that many sample preparation steps, such as filtration or fractionation require the MP reference particles to be suspended in order to accurately mimic environmental MP behavior (Hüffer et al., 2017), which renders them unsuitable as true reference materials for the evaluation of these steps. Another difficulty while preparing MP reference materials is the multitude of shapes that needs to be covered. This is of special concern if the particles are used for the development of image-based methods. In order to develop appropriate morphological characterization tools, particles for all shapes (fragments, spheres, films, fibers) need to be available preferably in the same sample, as a genuine sample would also display all morphologies at once. On the other hand there are experiments, such as toxicological investigations that require tailored methods to achieve specific morphologies, such as Cole (2016) for the production of fibers or Balakrishnan et al. (2019) for spheres. To summarize, MP and nanoplastic reference materials each have their own challenges, but both are desperately needed for further method development. The described shortcomings of current MP reference particles can be overcome by using sonication of polymers in alkaline suspensions for *in situ* fragmentation. Ultrasonic treatment of various polymers has already been used to broaden the size distribution of polymer powders in water (Price et al., 1995; Davranche et al., 2019). Furthermore, Balakrishnan et al. (2019) used a combination of dissolution of polyethylene (PE) in toluene via sonication with subsequent emulsification in water to create PE spheres in the subμm range (200–800 nm). They suggest that the same methodology could potentially be used on other polymers, as long as they can be dissolved in a volatile solvent. Davranche et al. (2019) fragmented environmental MP in water through sonication to generate nanoplastic. In this paper we propose, for the first time, the use of ultrasonication in alkaline conditions for the simple and controlled production of chemically aged MP particles from single use plastic items. To this end, we characterized morphological features, such as size distribution, shape, and surface properties, as well as chemical properties, such as purity and aging effects as well as the suspensibility in water. Therefore, by characterizing the produced fragments in all important features of MP, including homogeneity and stability, we aimed to establish them as suitable reference materials. The overreaching goal is to deliver an easy and reproducible protocol for the production of reference materials, as defined by NIST (2016), from the solid target polymer of the analysis. The described procedure was validated for polylactic acid (PLA), PET, and PS. The underlying mechanism was researched to explain the different degrees of reproducibility (PLA > PET > PS) of the method with respect to the polymer and to pinpoint possible pitfalls. This is important as the applied ultrasonic field plays a substantial role in the formation of the particles. This was one of the major challenges while performing reproducibility experiments, which required many fragmentation experiments and could only be overcome by testing the ultrasonic field (see Supporting Information) before starting the procedure. The final reproduction study was carried out by two different operators to ensure that the protocol is complete and comprehensible. Furthermore, the presented procedure was applied in proof of principle experiments to PE, polypropylene (PP), and polyvinylchloride (PVC) as well as polyamide (PA) to investigate if the method is generally applicable (see Supporting Information). Essentially any polymer that does not form a gel (like polyamides) in alkaline solutions can be fragmented through alkaline sonication but the hydrolizability and mechanical properties of the polymer influence size and number of the fragments.

## Materials and Methods

### Equipment Preparation and Avoidance of Contamination

A 1 M KOH stock solution (22.2 g KOH, Chemsolute Batch No 25.101811 dissolved in 400 mL MilliQ) was used throughout the experiments to prepare the sonication medium. All equipment was cleaned multiple times with water, isopropanol, and MilliQ water to minimize contamination of the samples. The reaction vessels were additionally sonicated in alkaline solution (1 M KOH ultrasonic bath, 15 min). Before sonication, the polymer squares were submerged in KOH (1 M, 3.75 mL, 30 s) to clear any attached organic matter. The solution was then diluted with MilliQ for the fragmentation. After sonication the samples were handled and filtered in a laminar flow box (EN 1822, Spetec GmbH).

### Fragmentation Through Sonication

The idea to use sonication as the fragmentation method arose from many accounts that suggested that sonication might lead to MP fragmentation, and therefore should be avoided for sample preparation. Very recently nanometer-sized fragments from sonication (5 days, MilliQ) were employed to test the binding abilities of lead to nanoplastic (Davranche et al., 2019). As the goal of our research was to provide aged and dispersible particles, polymer squares (1 cm^2^, ~30 mg) were sonicated (15 h at 35 kHz) under hydrolytic conditions (15 mL, 0.25 M KOH) to induce the formation of polar groups. Furthermore, the effect of the original fragment size on the produced size distribution was analyzed in a second experiment.

**Figure 1:**
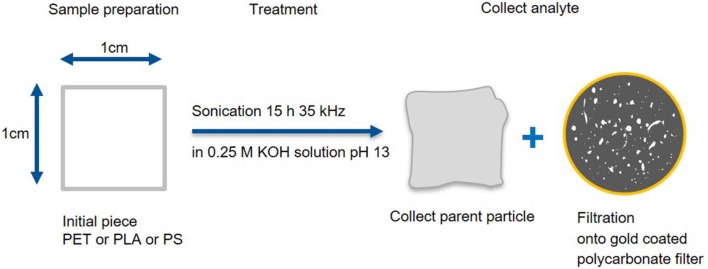
Fragmentation and work up scheme A. Production of small fragments and deposition on a gold-coated polycarbonate filter as well as recovery of the parent particle.

Fragmentation method A (Figure 1): Polymer squares (1 cm^2^, ~30 mg, PLA and PS from Activia yogurt cups and PET from a “Ja” water bottle) were sonicated (15 h at 35 kHz) in hydrolytic conditions (15 mL, 0.25 M KOH). The parent particle was collected and the leftover suspension was filtrated (in a laminar flow box onto a 25 mm diameter, 0.8 μm pore size, gold-coated polycarbonate filter, APC). To ensure that the produced MP was quantitatively transferred from the reaction vessel to the filter, all glass parts, that were in contact with the particle suspension, were rinsed with MilliQ water (about 30 mL) until no particles were visible under UV light (λ = 285 nm). Both the parent particle (before and after fragmentation) and the fragments on the filter were analyzed via Raman microspectroscopy and IR spectroscopy (detailed procedure in section “Characterization of the produced MP particles”). For each polymer the production and the subsequent measurements were repeated tree times by two different operators (*N* = 3). A smaller proof of principle study (only one replicate) was applied to PVC (from wide neck containers Rotilabo), PE (pellets from Huhtamaki), PP (yogurt cup “Penny Vanilla desert”), and PA (foil from Huhtamaki) to check whether the fragmentation method is also transferrable to other polymers. PVC was chosen, as it contains chlorine which can be detected by scanning electron microcopy coupled with energy dispersive X-ray spectroscopy (SEM/EDX) analysis to prove that the particles found in the nanometer range are indeed plastic particles produced by our method.

**Figure 2:**
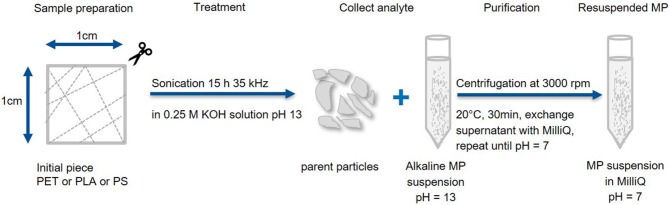
Fragmentation and work up scheme B. Production of large MP fragments with recovery of the parent particles. Particles smaller than 1 mm are washed and concentrated in MilliQ.

Fragmentation and work up method B (Figure 2): To generate particles in the entire MP size range (1 μm−1 mm) randomly cut polymer pieces (~30 mg) were sonicated (15 h at 35 kHz) in hydrolytic conditions (15 mL, 0.25 M KOH). Particles larger than the pipette opening (~1 mm) were collected for Raman and FTIR analysis, while the smaller fragments were collected from the alkaline suspension through centrifugation (3,000 rpm, 20°C, 30 min, Eppendorf 5804 R), removal of the supernatant and resuspension in MilliQ (pH = 7 was reached after 2 cycles).

### Characterization of the Produced MP Particles

The original polymer pieces were removed from the suspension and microscopically analyzed (5× and 20× magnification with z stacking 30 μm, Raman microscope *alpha 300*, WITec, Germany).

The fragments were collected on the filter and analyzed with a Raman microscope *alpha 300 (using TrueSurface, custom image stitching, z stacking, TruePower, PointViewer, and TrueMatch*, WITec, Germany) applying the following steps. Firstly, the planarity of the filter was checked (optional *TrueSurface* measurement Δz should be ≤ 30 μm) and an image of the filter was acquired (20× objective, 3 [db] Gain, 3% top illumination, 1/10 fps, 16,000 × 16,000 μm, 8,000 × 8,000 pixel, 30 μm z-stacking, by custom image stitching). The particles were localized (calculation of centers for Raman measurement) and morphologically analyzed (Feret's diameter min and max, area, ratio of Feret's diameter and percentage of area covered by particle in Feret's box for shape analysis) via image processing using *TUM-ParticleTyper* (Gaussian window-based detection, von der Esch et al. (submitted), preview in SI Figure 1). Subsequent Raman microspectroscopy (3 mW using *TruePower*, 532 nm laser, 2.5–20 s measurement time, 20× objective, inserting the determined coordinates by *TUM-ParticleTyper* via *PointViewer*) revealed the identity (database search with *TrueMatch*, for validation see SI Figure 2) of *n* ~ 500 randomly selected particles per filter as described by Anger et al. (2018). Combining these results an overall compound distribution (desired MP vs. contamination) and a compound-correlated size distribution could be determined. The error of measurement was calculated through the number of detected particles, the portion of measured particles and the percentage of MP found. For a summary of the procedure and measurement effort (see Table 1). Furthermore, the morphological data enabled the discrimination of particle shapes to determine predominant shapes and sizes of the examined polymers. Fibers were identified by *TUM-ParticleTyper* if one of two conditions was fulfilled: 1) dividing the product of min and max Feret's diameter by the area yields a ratio larger than four or 2) max Feret's diameter divided by min Feret's diameter is larger than two and the overall area of the particle is larger than 400 pixels. Spheres are characterized by a Feret's diameter min/max ratio close to one (0.9–1). These conditions were empirically established by analyzing images and varying the selection thresholds. The reproducibility (homogeneity) of the fragmentation was accessed by comparing the replicates (*N* = 3) for each polymer according to composition, mean particle size, size distribution, and particle to fiber ratio. Fibers were further characterized by manual Raman measurements and the stability of the sample was checked after storage (particles deposited on the filter stored for 9 months in glass Petri dishes).

**Table 1:** Measurement scheme for the Raman microspectroscopic analysis of microplastic, featuring the tools, exact measurement parameters, time, operator effort, and goal for each step.

	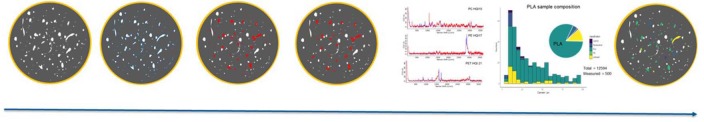						
**Steps**	**Take image of filter**	**Localize all particles**	**Select particles**	**Raman measurement**	**Chemical characterization**	**Data visualization**	**Overall performance metrics**
Tool	Image stitching + z stack	*TUM-ParticleTyper*	*TUM-ParticleTyper*	*TruePower + PointViewer*	*TrueMatch*	R-Script	–
Parameters	16,000 × 16,000 μm, 8,000 × 8,000 pixel, z stacking over 30 μm upwards starting from filter surface, Gain 3, Illumination 3%, exposure time 1/10, 20× magnification Darkfield mode	Min pixel 2, resolution 0.5 Detection in Raman mode	Random sampling of 500 particles per filter	532 nm laser, 3 mW, 2.5–20 s, 600 L/mm grating, 20× magnification	Correlation coefficient 2 components, HQI > 15,600–1,800 cm^−1^ Export as txt	Load *TrueMatch* and *TUM-ParticleTyper* result sheet and run script	Localization, quantification, morphological and chemical characterization of Raman active particles 10 μm−5 mm
Time	1 h	5–20 min depending on particle loading	30 s	~4 h	20 min	20 min	~6 h
Operator effort	20 min	2 min	–	1 h	20 min	5 min	~2 h
Goal	The entire filter surface should fit on the image	Particles and fibers of all sizes should be detected, verify by visual inspection	Minimize the amount of particles that need to be measured with reasonable margin of error	Minimize the time for a single measurement, but monitor the quality of spectra to get the best results in a reasonable time frame	Identification should be unambiguous, to ensure this, check low hit quality results 20–15 HQI	Merge morphological and chemical identification data to give a complete overview of the sample composition	Fast and easy detection of particulate matter on filter. Validation needs to be made for each type of sample individually

The current detection limit of our automated Raman setup used for this study is limited to particles larger than 5 μm (based on the smallest particles that yield identifiable spectra *TUM-ParticleTyper* with a 20× magnification objective, N.A. = 0.4). Using this setup, a quantitative analysis can be performed for particles larger than 10 μm preview in SI Figure 1. Particles in the low μm range and subμm range were alternatively characterized by manual Raman analysis (100× magnification objective, N.A. = 0.9) and SEM. This technique also allowed a special focus on the surface morphology of the particles. The SEM images were recorded on a *Sigma 300 VP* (Carl Zeiss AG, Germany) using a HD secondary electron detector. For sample preparation, the suspensions (10 μL) were dried on silicon wafer slices and could be imaged without the need for coating with metals due to the use of a FE Schottky cathode and low acceleration voltages (2–3 kV). EDX analysis was performed on PVC to ensure that the particles visible in the SEM images are in fact nanoplastics (*Quantax XFlash 6/60* detector, Bruker Nano GmbH, Germany). All analyses that can be performed on particles deposited on filters, such as automated Raman microspectroscopy and μ-FTIR were conducted on the same particles from one sample. SEM/EDX and manual Raman microspectroscopy for particles smaller than 5 μm requires an extremely smooth surface, therefore these were conducted on subsamples from the fragmentation, so that the same sample can be used but not the exact same particles.

To characterize the suspensibility of the produced particles (in 2.5 mL MilliQ) by UV-VIS (*Specord 250*, Analytik Jena, Germany) time series measurements were conducted (3 replicates for each polymer, 25 measurements in 25 min, λ = 250–800 nm, Δλ = 1 nm, slit 4 nm, speed 50.0 nm/s). A good signal could only be achieved by enriching the particle number in an aqueous suspension and then monitoring the transmission over time; therefore, fragmentation and work up method B was used.

The MP produced through fragmentation methods A and B by sonication should be similar, both chemically as well as morphologically to the MP present in the environment. To demonstrate this, attenuated total reflection Fourier transform infrared spectroscopy measurements (ATR-FTIR, *Nicolet 6700 FTIR*, 4 cm^−1^ spectral resolution) were conducted on the original polymer pieces before fragmentation (themed reference/pristine in subsequent sections) and on fragments larger than 1 mm. Particles < 1 mm were measured by μ-FTIR spectroscopy on *Agilent Cary 620* coupled to *Agilent Cary 670*, equipped with a 128 × 128 pixel focal plane array (FPA) detector. Measurements were performed in reflectance mode, using a 15× objective. Of each sample, 30 scans were recorded at a spectral resolution of 8 cm^−1^ within a spectral range from 3,700 to 810 cm^−1^.

## Results and Discussion

### Morphological Changes in Polymer Surface Due to Ultrasonic Degradation

**Figure 3:**
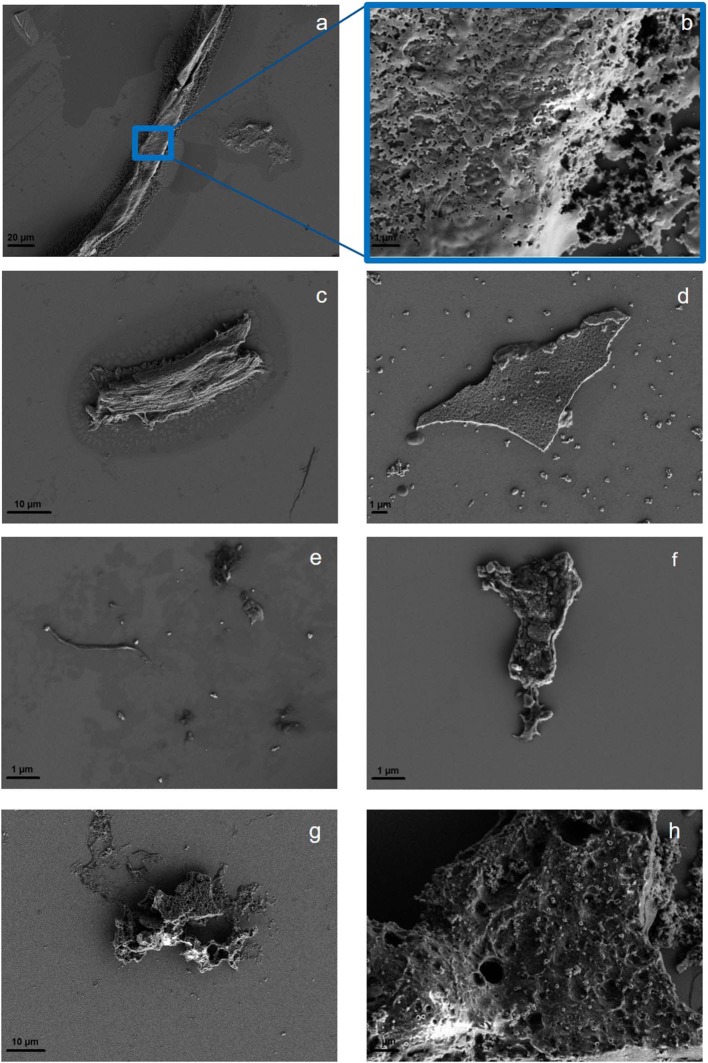
Surface morphology of PS showing a wide range of particle shapes: fiber **(a)**, with a closeup of the fiber surface **(b)**, irregular fragment **(c)**, and spheres and film **(d)**. PET fragments, fibers and spheres are shown in **(e)** with a closeup of the surface of a fragment **(f)**. Typical PLA fragment **(g)** with a closeup of the surface of a fragment **(h)**. More SEM images are available in SI Figures 6, 7.

The ultrasonic treatment of solid polymers leads to a changed morphology at the polymer surface, which is examined in the parent particles before and after sonication through optical microscopy (SI Figures 3A–C). For PLA, the sonication in MilliQ increases the cloudiness, making the original plastic square opaque. Although the edges of the square remain intact, it shrinks during the sonication process (1 cm^2^ to 0.57 ± 0.07 cm^2^). If sonication with KOH (0.25 M, pH = 13) is applied, the original square becomes cloudy and the edges are visibly eroded. Furthermore, holes in the surface appear. Changes in surface appearance that are observed for PET and PS are similar to PLA, but less intense (SI Figure 4). Only PLA shows a slight increase in cloudiness after sonication in KOH, while PET and PS do not turn opaque. Instead they show less particles attached to the surface and smoothed edges after treatment (SI Figure 4). Comparing the morphological changes induced by the fragmentation suggest different mechanisms. PLA as an ester might be strongly hydrolyzed, as well as mechanically worn, while PS and PET might be predominantly, but not exclusively mechanically fragmented, as subsequent ATR-IR analysis revealed through the appearance of OH, C=O, and COOH groups to all polymers (section “Comparison of Reference Particles with Environmental Microplastic by FTIR Spectroscopy”). To visualize the surface modification and to assess the shapes of the resulting particles, SEM was applied. Particle identity has been confirmed by SEM-EDX and Raman microspectroscopy (SI Figure 5). All polymers produce fragments, in irregular and spherical shapes as well as films and fibers. Furthermore, the surface is visibly eroded. As with the parent particles, PLA fragments seem to be most affected by the sonication, producing extremely swollen and porous particles. PS and PET show eroded surfaces as well. The smallest particles that have been visualized for all polymers are around 100 nm (Figure 3).

### Yield and Reproducibility of the Fragmentation

**Figure 4:**
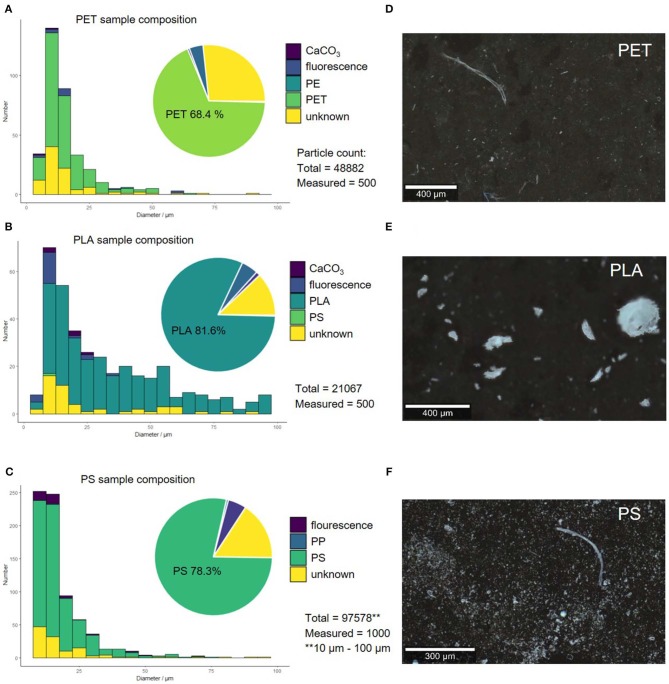
Compound correlated size distribution of replicate 1 by operator 1 for PET **(A)**, PS **(B)**, and PLA **(C)**. Microscopy images of polymer fragments produced through sonication in alkaline solution (replicate 1) PET **(D)**, PS **(E)**, and PLA **(F)**.

As can be seen in Figure 4 the polymer size (A–C) and shape (D–F) distribution is highly polymer-dependent. With the applicable microscopy set up, particles down to diameters of 5 μm could be analyzed, with the exception of PS—here, only particles larger than 10 μm could be analyzed through image processing, as there were too many particles to be processed below 10 μm (computation time exceeded 30 min).

PLA fragmented into 1.1 × 10^4^-2.1 × 10^4^ comparatively large particles, which are mostly fringy irregular fragments. PET and PS yielded 1.8 × 10^4^-7.1 × 10^4^ and 9.2 × 10^3^-2.1 × 10^5^ smaller more jagged particles, respectively. The fragmentation of PLA and PET lead to reproducible results, as the number of fragments is within the same order of magnitude for all replicates. The fragmentation of PS is less reproducible, as we achieved fragment counts that are several orders of magnitude apart. This might be caused by the fragmentation mechanism through sonication in alkaline conditions, which relies on two parameters: Hydrolysis, which is systematic and controllable through pH and mechanical strain, which is systematic, but less controllable because it is dependent on the ultrasonic field. PLA is easily hydrolysable, while PET and PS are increasingly less hydrolysable and therefore the fragmentation mechanism must be more dependent on the less controllable mechanical strain and radical decay (see section “Mechanistic Implications for the Degradation of Solid Polymers by Sonication”).

The sample composition, however, was very similar within the replicates and among the different polymers. In all samples also polycarbonate particles from the filter were found (~15–25%). These were removed from the compound distribution, as they are artifacts originating from the filtering material itself and are not present in the generated suspension. After removing these artifacts from the analysis, the composition of the samples created through sonication in alkaline solution were analyzed. The original polymer was the predominant component (~68.4–81.6% depending on the polymer). All samples also contained a portion of particles that could not be classified, as the spectra were too noisy (HQI < 15 and manual identification failed), displayed only background signal or showed spectra for which no library match could be found (~12.2–26.8%). Only a very small number of particles (4.0–5.1%) showed too strong fluorescent backgrounds to be analyzed. Contamination with other polymers and CaCO_3_ was negligible (0.6–1.2%). The Raman spectra of the polymers showed no signs of aging in the form of additional bands, which is in accordance with prior aging experiments (Lenz et al., 2015; Cai et al., 2018) (see SI Figures 8–10 for Raman spectra before and after fragmentation).

**Figure 5:**
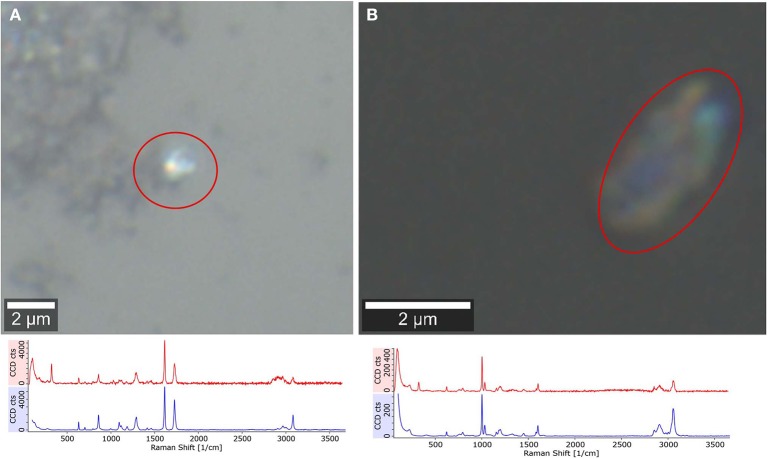
Raman microspectroscopic analysis of plastic fragments, confirming the formation of low μm range particles through sonication. PET (**A**, reference blue, particle spectrum red), PS (**B**, reference blue, particle spectrum red).

Furthermore, 10 μl of sample were deposited on a CaF_2_ substrate to measure selected particles ~2 μm (*n* = 30) to confirm the formation of small plastic particles through sonication, which was suggested but not chemically proven by Davranche et al. (2019). In Figure 5 the smallest detectable particle through Raman microspectroscopy (100× magnification objective, N.A. = 0.9) for PS and PET is shown. This does not prove that all particles of this size originate from the plastic material but confirms the formation of small plastic particles through sonication.

**Figure 6:**
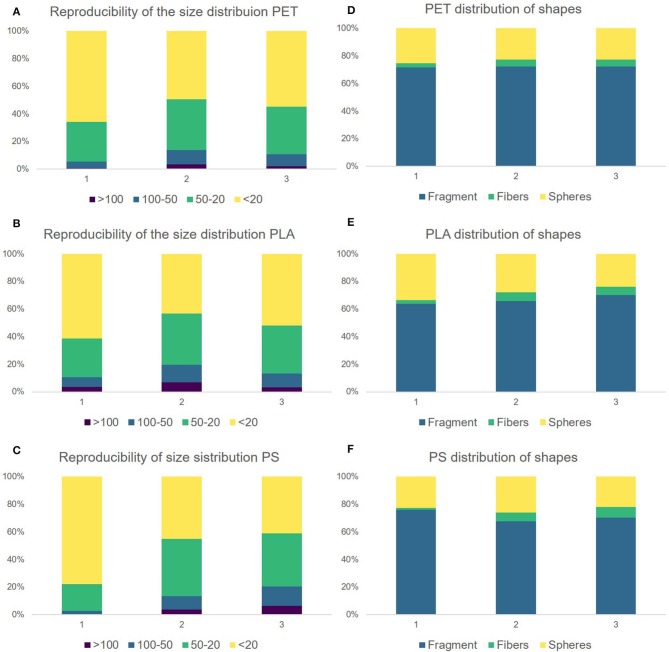
Comparison of the replicates 1–3. Replicate 1 was produced by operator 1 and replicates 2 and 3 were produced by operator 2. Reproducibility of the size distribution for PET **(A)**, PLA **(B)**, and PS **(C)**. Distribution of particle shapes for PET **(D)**, PLA **(E)**, PS **(F)**. The corresponding data can be viewed in SI Table 1.

The reproducibility of the size distribution and of the shape variation was tested by replication (*N* = 3). Figure 6 shows that there is variation in between the replicates but overall the procedure leads to comparable results. The dominant size fraction is <20 μm, which accounts for half of the particles of PLA (52.2 ± 9.0%), PET (56.55 ± 8.3%), and PS (54.7 ± 20.2%). We have, however, noted that there is a dip in the 5–10 μm size class, were we would have expected an increase in particle number. This suggests to us that although the measurement of particles in the size range 5–10 μm is possible as identifiable Raman spectra can be measured, this class may not be quantitatively represented by the method applied as indicated in von der Esch et al. (submitted). The second largest fraction is 20–50 μm for PLA (33.3 ± 4.7%), PET (33.4 ± 4.1%), and PS (33.0 ± 12.0%). Particles larger than 50 μm are present but make up only a small portion for PLA (14.5 ± 3.8%), PET (10.0 ± 3.5%), and PS (12.3 ± 7.3%). Although fragments in the shape of irregular fragments, fibers as well as spheres and films were found by optical microscopy and SEM, irregular fragments are the predominant shape (66.4–72.1%) in all replicates across all polymers. The second major fragment shapes that occur are spheres (23.5–28.4%) followed by fibers (4.2–5.3%). Since our automated categorization is based on the comparison of diameter ratios and areas, we cannot further characterize into films with our current program. Fibers were additionally characterized via manual Raman microspectroscopy to ensure that they truly originate from the fragmentation of the polymer parent particle (see SI Figures 11–14).

### Size Distribution Comparison to Environmental MP Particles and Other Reference Materials

A major challenge in MP production from both milling and sonication is control over particle size distribution. For the (cryo)milling, the size distribution seems to depend on the temperature, loading, and type of polymer (Eitzen et al., 2018; Kuhn et al., 2018). The major factors of influence on MP formation through sonication will be reviewed in detail in the following sections. Both preparation methods were compared to aging experiments by Lambert and Wagner (2016) and show that all MP production methods yield different size distributions. While milling produces large particles predominantly [50–100 μm (Eitzen et al., 2018) and 500 μm−2 mm (Kuhn et al., 2018)] sonication rather leads to smaller particles (average Feret's diameter, μ = 30.23 ± 12.14 μm for PS, μ = 32.04 ± 6.53 μm for PLA and μ = 26.56 ± 5.23 μm for PET). The average size of the particles produced through sonication is much closer to the average particle sizes relevant in the environment (Anger et al., 2018) and in weathering studies (~99% of PLA, PS and PET particles are in the size range 0.6–18 μm) (Lambert and Wagner, 2016).

If extremely small particles are desired, some alternative procedures to the sonication method presented here are available. PS nanoparticles (125–437 nm) can be produced by blending in a food processor (Ekvall et al., 2019). For PET, laser ablation delivers nanoplastic (Magri et al., 2018). If specific shapes are the target of the production process, fibers (40–100 μm length) from nylon, PET, and PP can be produced by using a cryogenic microtom (Cole, 2016) and spheres in the nm range are commercially available for PS.

Our method provides an easy production of small MP fragments (1 μm−1 mm) but is highly dependent on the ultrasonic field. While subμ particles were also present in the suspensions produced through sonication as demonstrated by SEM/EDX analysis, additional analysis via asymmetric field flow fractionation or centrifugal field flow fractionation and possibly staining for chemical characterization is necessary to quantify the particles formed in this size range (Schwaferts et al., 2020).

### Mechanistic Implications for the Degradation of Solid Polymers by Sonication

The effect of sonication is usually tested on polymers in various organic solvents for molecular weight tuning (Gogate and Prajapat, 2015). The mechanistic picture is brought forward that sonication creates cavities in the liquid medium, which release energy during cavity collapse resulting in local pyrolytic conditions [about 5,000 K, 2,000 atm (Leonelli and Mason, 2010)] and the release of radicals. In water, OH^.^ and H^.^ radicals are formed, which create hydrogen peroxide (H_2_O_2_), thus providing oxidizing conditions. At the molecular level, in addition a rapid movement of solvent molecules is induced that cannot be followed at the same scale by the macromolecules in the solvent. Thus, friction is created which causes strain and ultimately bond breaking in the macromolecules. The chains are preferentially split at transitions between amorphous and crystalline regions (Price and Smith, 1991). In our case, these conditions are used to induce polymer scissions through physical breakage as well as radical polymer degradation. For the samples where KOH (0.25 M, pH = 13) was added, additional OH^−^ ions are available to provide strong hydrolysis/oxidation conditions for the newly split polymers, which lead to a strong increase in the number of detected fragments. The focus of our investigation was PLA, PET, and PS, where we conducted reproducibility analyses for the fragmentation. We also tested PE, PP, PVC, and PA to prove that the procedure is applicable to any polymer. The results are shown in the supporting information (SI Table 2), as these were singular experiments and reproducibility can therefore not be accessed for PE, PP, PVC, and PA at this point in time.

It is important to note that all experiments were conducted in an ultrasonic bath which has the disadvantage that the fragmentation is not necessarily reproducible. Each ultrasonic bath produces its own inhomogeneous field (Jenderka and Koch, 2006). To make this procedure reproducible, the field parameters, which are related to the cavitation effects, need to be investigated prior to particle fragmentation (for details see SI Figure 15). Alternatively, a more sophisticated reactor design may be resorted to, which could also lead to better results and higher reproducibility. We decided to follow the first approach because we would like to enable anyone to repeat our fragmentation without the need for additional equipment.

Since not all laboratories will have the same ultrasonic bath at hand, the parameter-effect relationships are important to consider. For a conclusive review on current sonochemical research (reactor geometry, size, and solvent effects) we refer to Jenderka and Koch (2006), Liu et al. (2013), and Gogate and Prajapat (2015).

### Analysis of Suspensibility and Sedimentation Rates by UV-VIS Spectroscopy

A common problem with (cryo)milled particles is their static charge, which prevents the suspension in water. Eitzen et al. (2018) even reported adhesion to glass walls for PS fragments from cryomilling, which increased with decreasing size. However, due to their density (1.04 g/cm^3^) PS fragments should sediment in water, as is observed, especially in non-stirred PS suspensions (Hüffer et al., 2017). In order to alleviate the suspensibility issue usually one of two paths are chosen: (i) Suspension with a surfactant, which renders the sample unsuitable for toxicological testing, or (ii) oxidative treatment to modify the fragment surface, creating polar groups. For cryomilled particles both treatments require an additional processing step.

**Figure 7:**
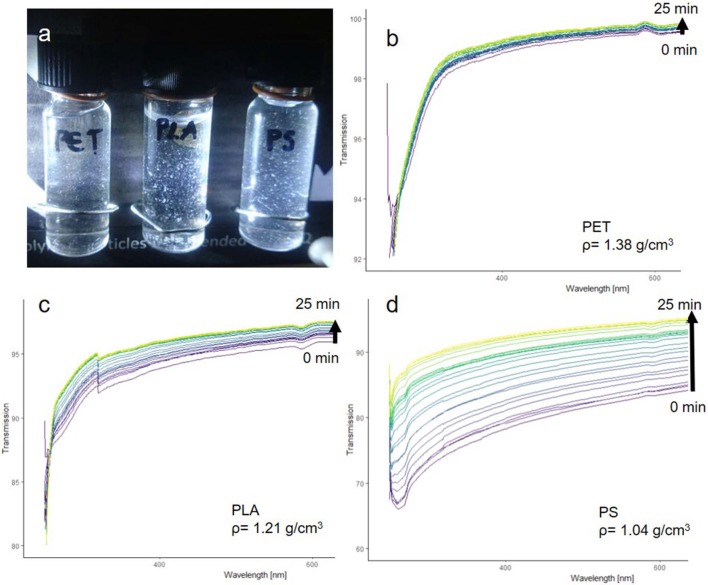
PET, PLA, and PS fragments suspended in pure MilliQ water **(a)**. UV-VIS transmission spectra of PET **(b)**, PLA **(c)**, PS **(d)**.

The fragments produced through sonication were suspended in pure MilliQ (Figure 7a) to test the suspensibility and sedimentation rate. Particles were observed to remain suspended without visible adhesion to the walls of the centrifuge tube. All suspensions were examined with UV-VIS, as suspended particles will absorb light leading to a low transmission signal. With increasing sedimentation, the transmission signal should increase and level off at 100%, which is the transmission of MilliQ without suspended particles.

The UV-VIS analysis (Figures 7c,d) shows that the fragments can be well-suspended and that they sediment according to their density. PET particles had the fastest sedimentation rate, while PLA particles sedimented slower and PS particles showed the slowest sedimentation rate. The suspensibility is likely a result of the *in situ* fragmentation in an alkaline solution, which introduces hydrophilic groups into the polymer surface while the fragments split off from the parent particle (see section below “Comparison of reference particles with environmental microplastic by FTIR spectroscopy”).

Thus, the produced fragments showed the desired sedimentation properties without the need for a surfactant, making them suitable for toxicological testing and validation of recovery rate experiments for sample preparation as well as for detection of MP.

### Comparison of Reference Particles With Environmental Microplastic by FTIR Spectroscopy

The goal was to create reference particles, which mimic the properties of secondary MP formed in the environment through weathering. Therefore, we compared the spectra of our *in situ* aged fragments to reference polymer spectra and the spectra acquired by Scott Lambert and Martin Wagner in their 112 days weathering experiment (Lambert and Wagner, 2016).

**Figure 8:**
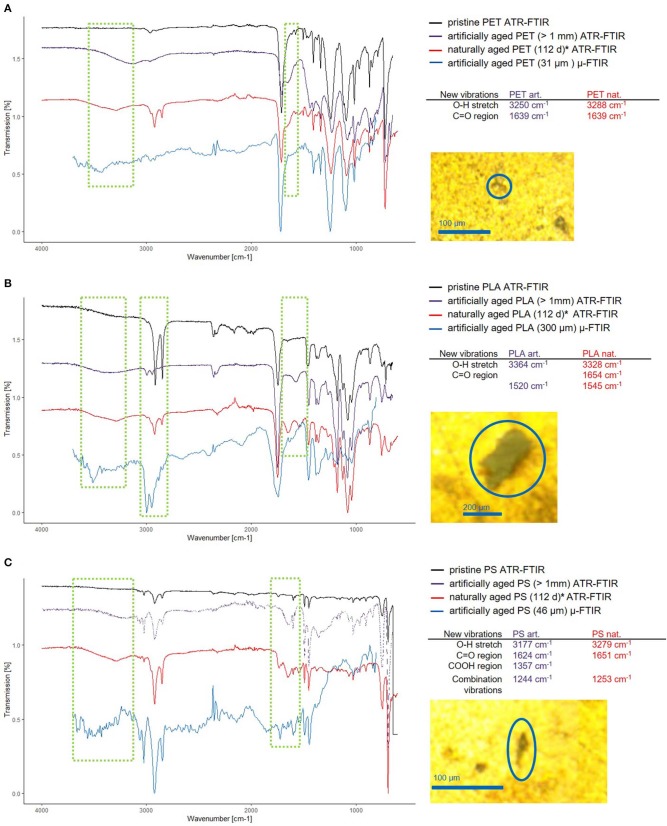
Comparison of the new vibrations in artificially and naturally aged polymers. Thanks to Scott Lambert and Martin Wagner, who provided the data for 112 days aged MP (Lambert and Wagner, 2016) (marked with *), a comparison between naturally and artificially aged MP was possible. The ATR-FTIR spectra for pristine (black) artificially aged (purple) and naturally aged (red) and reflectance μ-FTIR spectra of small artificially aged MP (blue) are shown for PET **(A)**, PLA **(B)**, and PS **(C)**, wherein the regions of interest are marked (green squares). The positions of the bands corresponding to the newly formed functional groups of the ATR-FTIR measurements are listed and the microscopy images of the particles analyzed are displayed to the right side of the spectra.

Fragments larger than 1 mm of all three tested polymers (PLA, PS, and PET) showed significant changes in their FTIR spectra (Figures 8A–C).

The most pronounced changes are exhibited by PLA, which shows additional bands (3,364 cm^−1^, O–H and 1,520 cm^−1^, C=O) and shifting C-H stretching vibrations (blue shift, 2,916 cm^−1^ → 2,993 cm^−1^ and 2,848 cm^−1^ → 2,930 cm^−1^). Interactions of the C–H group with neighboring O–H groups cause the band shift of the C–H stretching vibration (Schmidt et al., 2006). This and the emergence of a band at 3,364 cm^−1^ shows either the formation of O–H groups through hydrolysis or the intercalation of water molecules into the polymer structure. Here, a combination of both is likely, as the appearance of COOH introduces electrostatic interactions which lead to stronger swelling of the polymer (Proikakis et al., 2006). The overall shifts in intensity hint at a changed ratio of crystalline/amorphous structure within the polymer (Bower and Maddams, 1989). When comparing the spectra of the artificially and naturally aged PLA, we observe that the resulting spectra have similar new bands, leading to the conclusion that sonication under hydrolytic conditions is an effective method to artificially age polymers to produce reference materials.

PET shows changes in relative band intensity and additional bands (3,250 cm^−1^, O–H and 1,639 cm^−1^, C=O). The additional band at 3,250 cm^−1^ is indicative of the formation of O–H groups (Zhang et al., 1996), as PET does not absorb water well (Venkatachalam et al., 2012), but is known to hydrolyze at high temperatures (*T* = 80–200°C) (Achilias and Karayannidis, 2004), which are easily reached through sonication as collapsing cavities create local pyrolytic conditions. In natural environments photo-oxidation and hydrolysis are the driving forces for PET degradation (Gewert et al., 2015) which should lead to similar results as the proposed sonication provides radicals and alkaline conditions. When comparing the spectra of the artificially and naturally aged polymers, we again see very similar modifications.

PS shows changes in band intensity and additional bands (3,177 cm^−1^, O–H; 1,624 cm^−1^, C=O as well as 1,357 cm^−1^, COOH and 1,244 cm^−1^), which are in accordance with the appearance of new bands found in naturally aged PS. The main degradation mechanism in a natural environment is photodegradation for PS, which in the presence of oxygen may introduce C–O bonds of various kinds leading to crosslinking and the formation of ketones (Yousif and Haddad, 2013).

For all additional bands it has to be said, that they cannot exactly correspond to the bands found in the naturally aged polymers, as the newly formed O–H, C=O, and COOH groups must have different interaction partners, which determine the exact position of the band within the group range.

To investigate if smaller polymer fragments display the same aging behavior as the large fragments, they were analyzed by μ-FTIR spectroscopy in reflectance mode. The number of measured particles was 10 for PLA and PET and 5 for PS (spectra available in SI Figures 17–19). Typical examples of MP spectra are given in Figures 8A–C. It is important to note that ATR and reflectance IR have different axial resolution, so that ATR will give spectra representing mostly the surface chemistry (~1–2 μm, axial resolution, see calculation in Supporting Information) of the particle. Reflectance IR will pass through particles with rough surfaces (~2.6 μm at 3,785 cm^−1^ to 20 μm at 500 cm^−1^, axial resolution, see calculation in SI Figure 16) and yield a spectrum describing the layer close to the surface of the particle at high wavenumbers but showing also the bulk properties of the particle at low wavenumbers.

Therefore, as it was the case in the ATR measurements, we can observe the C-H shift (2,916 cm^−1^ → 2,997 cm^−1^ and 2,848 cm^−1^ → 2,947 cm^−1^) inherent to the surface modification for the PLA sample (see Figure 8B). In addition, the broad O–H stretch band at 3,509 cm^−1^ is present. The C=O band of pristine PLA (1,744 cm^−1^) now shows a shouldering toward lower wavenumbers, resulting from the introduction of new C=O groups from the ultrasonic treatment. However, this is only visible as a shoulder, not as a free band as the light penetrates up to 6 μm deep and therefore gives more information on the bulk material than on the particle surface.

Regarding the PET sample (Figure 8A), the μ-FTIR measurements revealed the same spectra alterations as the ATR measurements. A C=O band shouldering at 1,623 cm^−1^ appeared as well as a very broad O–H stretch band at 3,420 cm^−1^.

In the PS sample, only the O–H band (3,501 cm^−1^) appeared, whereas the other changes observed using ATR-FTIR do not show (Figure 8C).

The reflectance μ-FTIR measurements confirm that the surface modifications seen in the large particles also appear in the small fragments.

Therefore, our results suggest that if aged MP fragments are required for experimental work or validation, a simple 15 h sonication in alkaline conditions can provide materials that mimic not only the sedimentation properties, but also surface chemistry of aged MP.

## Conclusion

Currently available MP reference particles (1 μm−1 mm) are usually produced by grinding. To this end special cryomills are needed. This equipment is not accessible in every laboratory and thus decouples the manufacturers from the users, which makes fast method development difficult. In contrast, the ultrasonic-based method brought forward here makes it possible to produce reference particles in every chemical laboratory. These reference materials are already suspended during their formation process and can be resuspended in pure MilliQ (pH = 7). Furthermore, they have hydrophilic groups on the surface due to the production process, so that realistic environmental MP can be emulated. In addition, the different sizes and shapes help in the further development of image recognition methods used in Raman microspectroscopy and μ-FTIR spectroscopy to determine the measuring points.

The approach presented here delivers a mixture of particle shapes, which consists of predominantly irregular fragments. If the research question demands the investigation of a distinct shape, e.g., toxicological studies, monitoring the impact of fibers, an alternative method needs to be used. Furthermore, we would like to point out that the reproducibility was tested for PLA, PET, and PS and each laboratory employing the presented method will need to check the reproducibility with their equipment and with their polymers of choice. We have shown that it is possible to achieve reproducible results and have pinpointed the main influencing factors on the reproducibility namely the ultrasonic field, the mechanical properties of the polymer, as well as the hydrolizability. In order to achieve the comprehensive characterization and quantification of MP reference materials a protocol including Raman microspectroscopy, ATR-IR, μ-FTIR, UV-VIS, SEM, and EDX was presented, which can be applied to any MP sample. We have shown by qualitative analysis that sonication also produces particles in the subμ plastic range. If these particles are to be used in future experiments, further quantitative analyses will be required.

## Outlook

Future experiments will include an upscaled procedure for the production of aged MP reference material. A possible path might include the (cryo)milling of polymers followed by the sonication (at alkaline conditions), thus enabling a high yield and the *in situ* generation of small, aged and suspensible particles. In addition to the fragmentation, the *in situ* coating of MP with humic substances could provide a helpful model to mimic the environmental adsorption of dissolved organic carbon and thus check the detectability of particles under environmental conditions. This is also a very important research gap so far, as it is often stated that Raman microspectroscopy is limited by the attachment of organic matter to the analyte inducing fluorescence and preventing the detection of particles. Even though it is extremely important to know the detection limits of a method, currently there is no systematic examination of this matter. With our optimized scheme for simple and reproducible generation of reference particles that can be adopted by laboratories around the world, using their polymers of interest, we hope to enable easier and more detailed method development and validation, as well as toxicological testing for future studies.

## Data Availability Statement

The raw data supporting the conclusions of this article will be made available by the authors, without undue reservation, to any qualified researcher. We are delighted to respond to data requests as the full data set for all experiments for our reference particle study contains a total of 300 GB (including all experiments necessary to get to the point where the procedure is reproducible) mostly consisting of classified spectra (Raman, IR UV-VIS, EDX) and images (Optical microscopy, SEM).

## Author Contributions

EE, ME, and NI designed the experiments. EE implemented the idea for the production of secondary MP, carried out the fragmentation, *TUM-ParticleTyper* Raman microspectroscopy, ATR-FTIR, and UV-VIS experiments, and validated the procedures. Reproducibility was tested by ML, under the supervision of EE, ME, and NI, by replicating the fragmentation and *TUM-ParticleTyper* Raman microspectroscopy analysis. The *TUM-ParticleTyper* was developed, optimized, and validated by AK, EE, and NI. The SEM and EDX analyses were carried out by CS. JW, KG, and TH investigated the aging effects on the single particle level with μ-FTIR spectroscopy. All authors discussed the results and contributed to the final manuscript.

### Conflict of Interest

The authors declare that the research was conducted in the absence of any commercial or financial relationships that could be construed as a potential conflict of interest.
