# Supplementary material for: Simple Generation of Suspensible Secondary Microplastic Reference Particles via Ultrasound Treatment
Source: Front Chem. 2020 Mar 18;8:169. doi: 10.3389/fchem.2020.00169 (PMC7093332; doi:10.3389/fchem.2020.00169)
Supplement: Supplementary file 1 [file Data_Sheet_1.PDF]

Supplementary Material

Simple Generation of Suspensible Secondary Microplastic Reference Particles via Ultrasound Treatment

Elisabeth von der Esch[a], Maria Lanzinger[a], Alexander J. Kohles[a], Christian Schwaferts[a], Jana Weisser[b], Thomas Hofmann[b], Karl Glas[b], Martin Elsner[a], Natalia P. Ivleva[a]*

[a]Institute of Hydrochemistry, Chair of Analytical Chemistry and Water Chemistry, Technical University of Munich, Munich, Germany

[b]Chair of Food Chemistry and Molecular Sensory Science, Technical University of Munich, Freising, Germany

*** Correspondence:**
Natalia P. Ivleva
natalia.ivleva@tum.de

1 Supplementary Data

Preview *TUM-ParticleTyper*

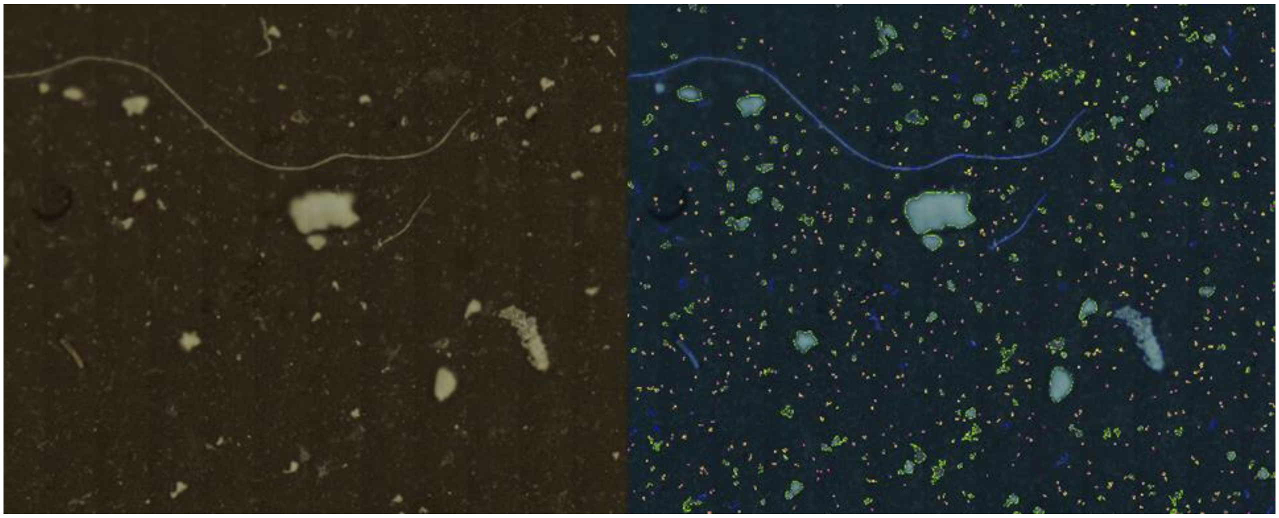

#	cx	Cy	area	diameters_min	diameter_max	classification
1	-1662.6	-7995.5	122.5	10.0	21.0	Particle
2	-3947.9	-7994.6	128.5	10.0	16.0	Particle
3	3251.4	-7992.7	279.5	16.0	27.0	Particle
4	1118.1	-7993.4	191.0	17.0	18.0	Particle
5	-2074.1	-7991.2	381.0	18.0	27.0	Particle
6	4406.7	-7991.4	487.0	20.0	37.0	Particle
7	792.5	-7992.6	464.5	20.0	41.0	Fiber
8	3341.5	-7999.0	1402.5	21.0	135.0	Fiber
9	2128.4	-7989.4	320.0	21.0	21.0	Particle
10	-2991.3	-7991.1	1897.0	21.0	167.0	Fiber
11	-4613.4	-7989.4	148.0	13.6	22.9	Particle
12	-4093.1	-7988.9	319.0	24.0	25.0	Particle
13	1956.5	-7990.4	935.0	26.0	66.0	Fiber
14	1438.4	-7988.0	486.5	26.0	26.0	Particle

Figure 1: Example of particle and fiber detection using TUM-ParticleTyper, left original image, right processed image. Particles are marked in green, fibers in blue. The red and blue dots mark the center points calculated for each particle. An example of a morphological characterization for particle and fiber detection using the TUM-ParticleTyper can be seen at the bottom. Here the particle number (#), the center coordinates (cx, cy), the area (area), the Feret's diameters (diameters_min, diameters_max) and the classification into fiber or particle (classification) are displayed. All data were reprocessed with the latest TUM-ParticleTyper version July 2019 to ensue best and consistent results (E. von der Esch, A. Kohles et al. submitted [1]).

Validation of Database Identification with *TrueMatch*

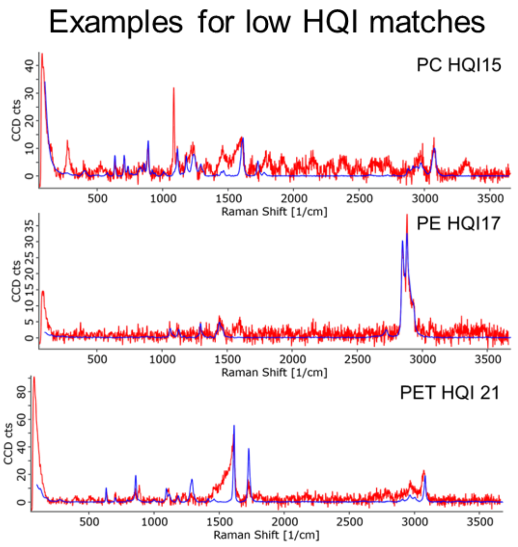

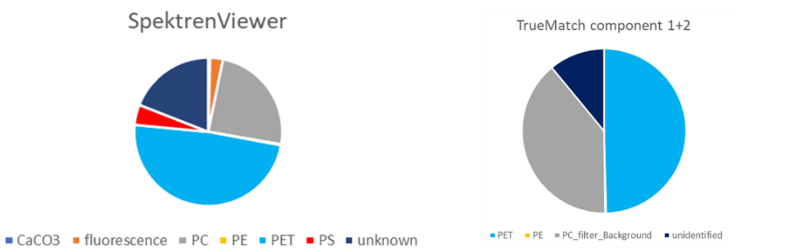

Visual assessment operator 1

classification	absolute	%
CaCO3	2	0.4
Fluorescence *	14	2.8
PC	122	24.5
PE	1	0.2
PET	242	48.6
PS**	22	4.4
unknown	95	19.1
**Sum**	**498**	**100**

Correlation coefficient 2 components
HQI > 15, 600 -1800 cm^{-1}

classification	absolute	%
PET	248	49.6
PE	1	0.2
PC_filter_Background	196	39.2
unidentified	55	11
**Sum**	**445**	**100**

*Fluorescence can not be classifier for a database search, it was only used in manual spectral analysis to check if even pristine MP samples show fluorescence
**Misidentification by operator was PC + band over 1000 resulting from salt impurities

Figure 2: All spectra from Replicate 1 were manually classified by operator 1 and then reprocessed with TrueMatch using a custom database. On the left examples for low HQI matches are displayed. HQI > 15 was found to be the lowest acceptable value all classifications below this value were marked as unidentified. All TrueMatch identifications below HQI = 20 should be checked before continuing the analysis, which is why the TureMatch analysis takes 20 min, the actual runtime of the program is ~ 30 sec for 1000 spectra. A comparison of the spectral identification of operator1 (middle) vs. True match (right) shows that the identification with both methods leads to comparable results.

Morphological Changes in Polymer Surface Due to Ultrasonic Degradation

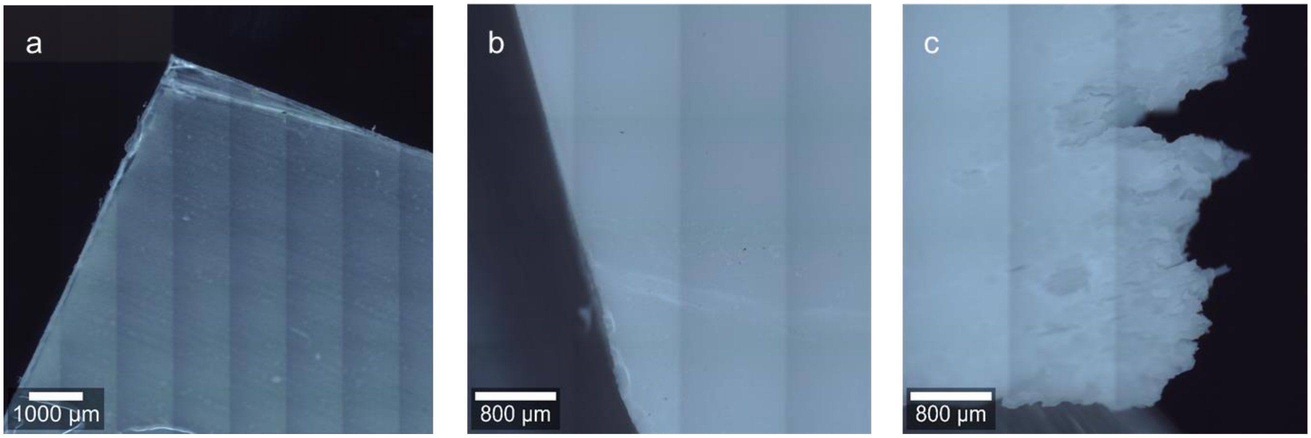

Figure 3: Surface of PLA square, before sonication (a), after sonication with MilliQ (b) and KOH (c) recorded on a Witec alpha 300 Raman microscope 20× magnification.

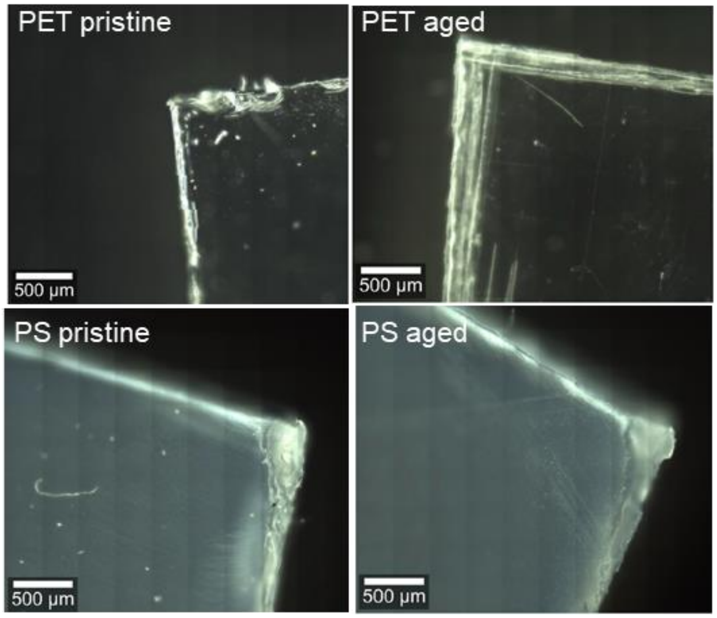

Figure 4: Surface of the polymer parent particle, before sonication and after sonication in alkaline solution recorded on a Witec alpha 300 Raman microscope 20× magnification.

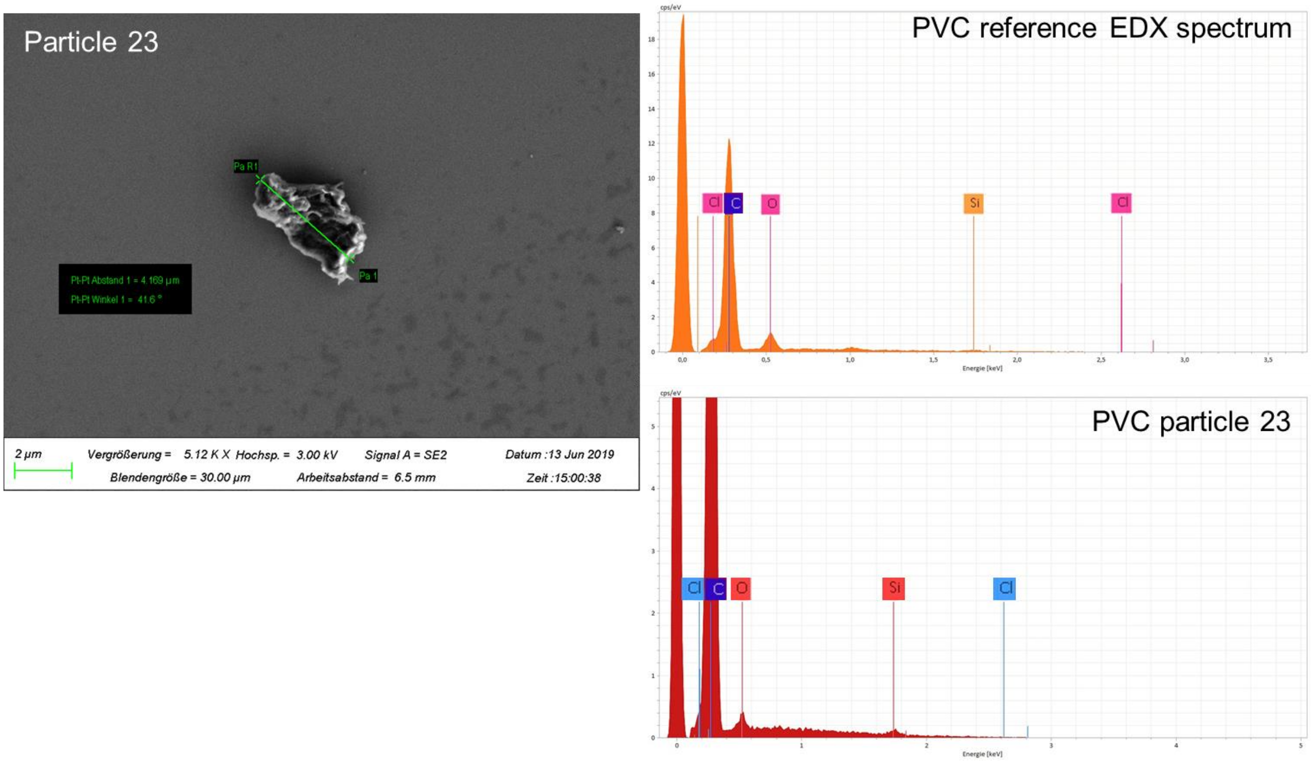

Figure 5: Example for a SEM/EDX analysis of PVC microplastic particles.

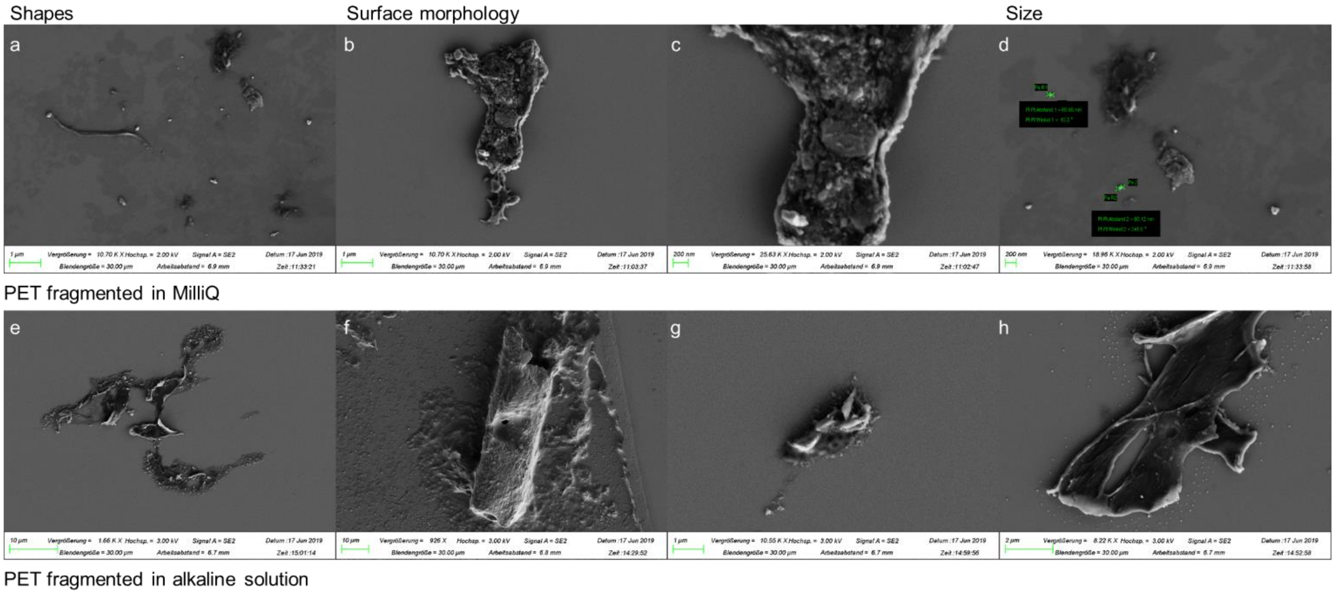

Figure 6: Surface morphology changes by fragmentation in pure MilliQ and KOH.

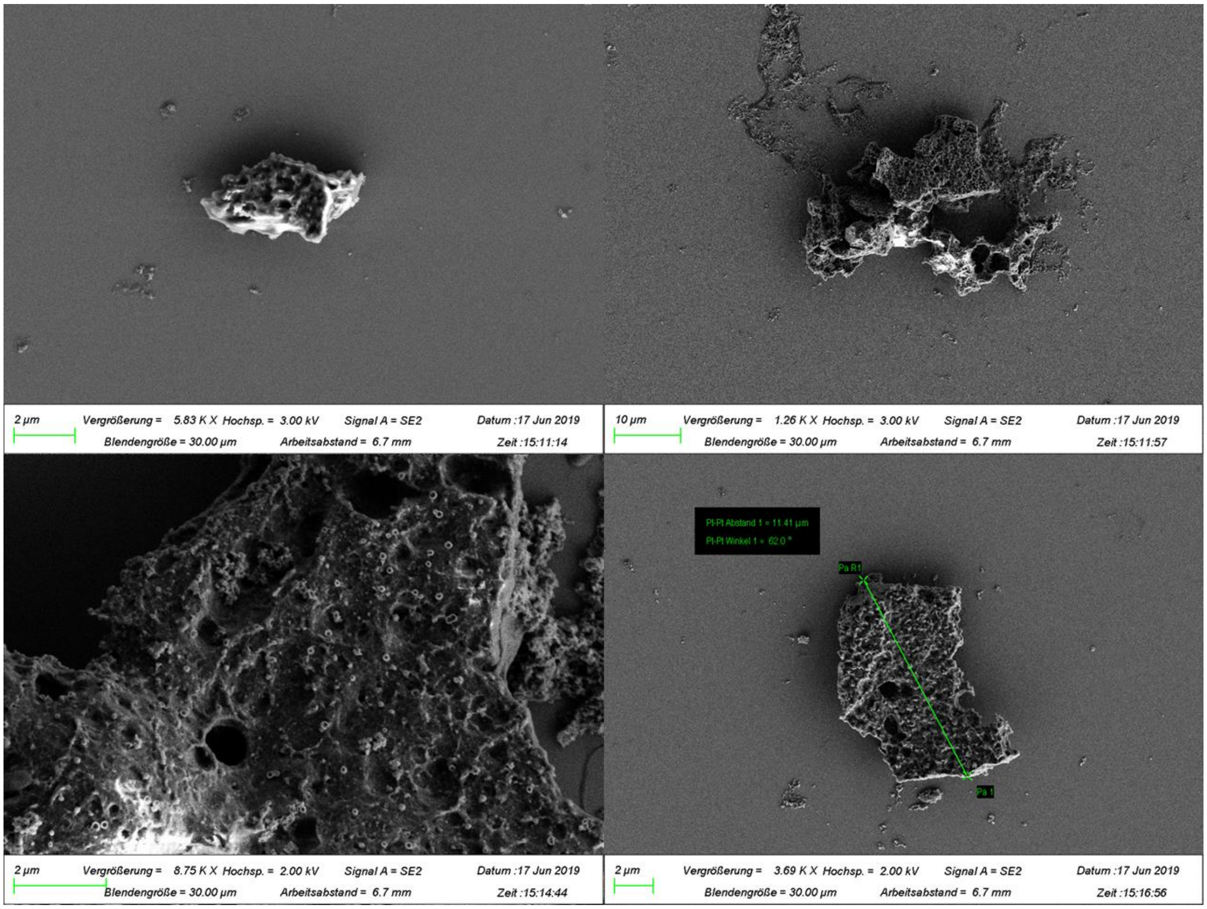

Figure 7: Surface modification of PLA.

Yield and Reproducibility of the Fragmentation

Characterization of the starting materials

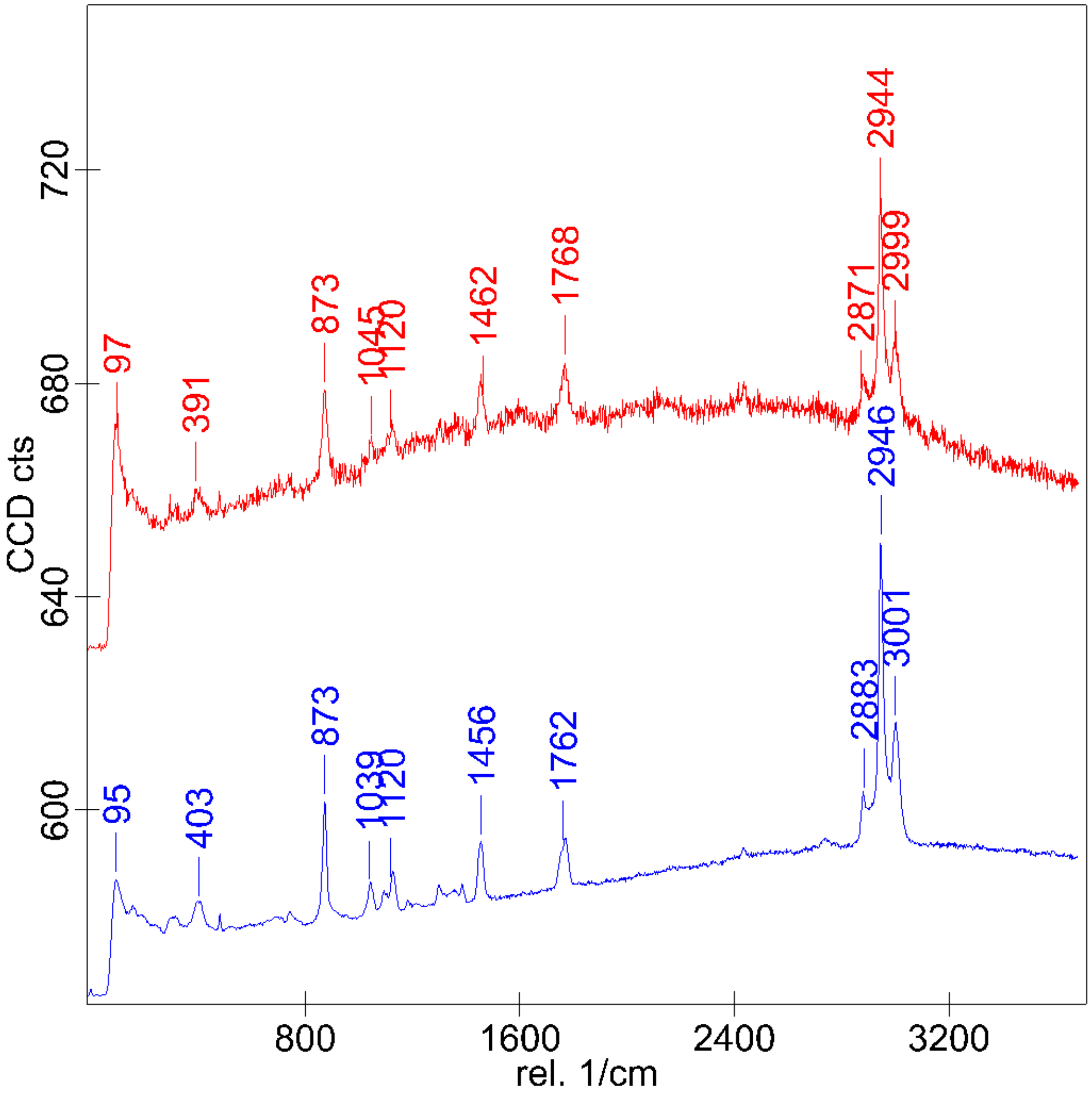

Figure 8:Exemplary spectrum of PLA starting material (blue) fragmented PLA (red).

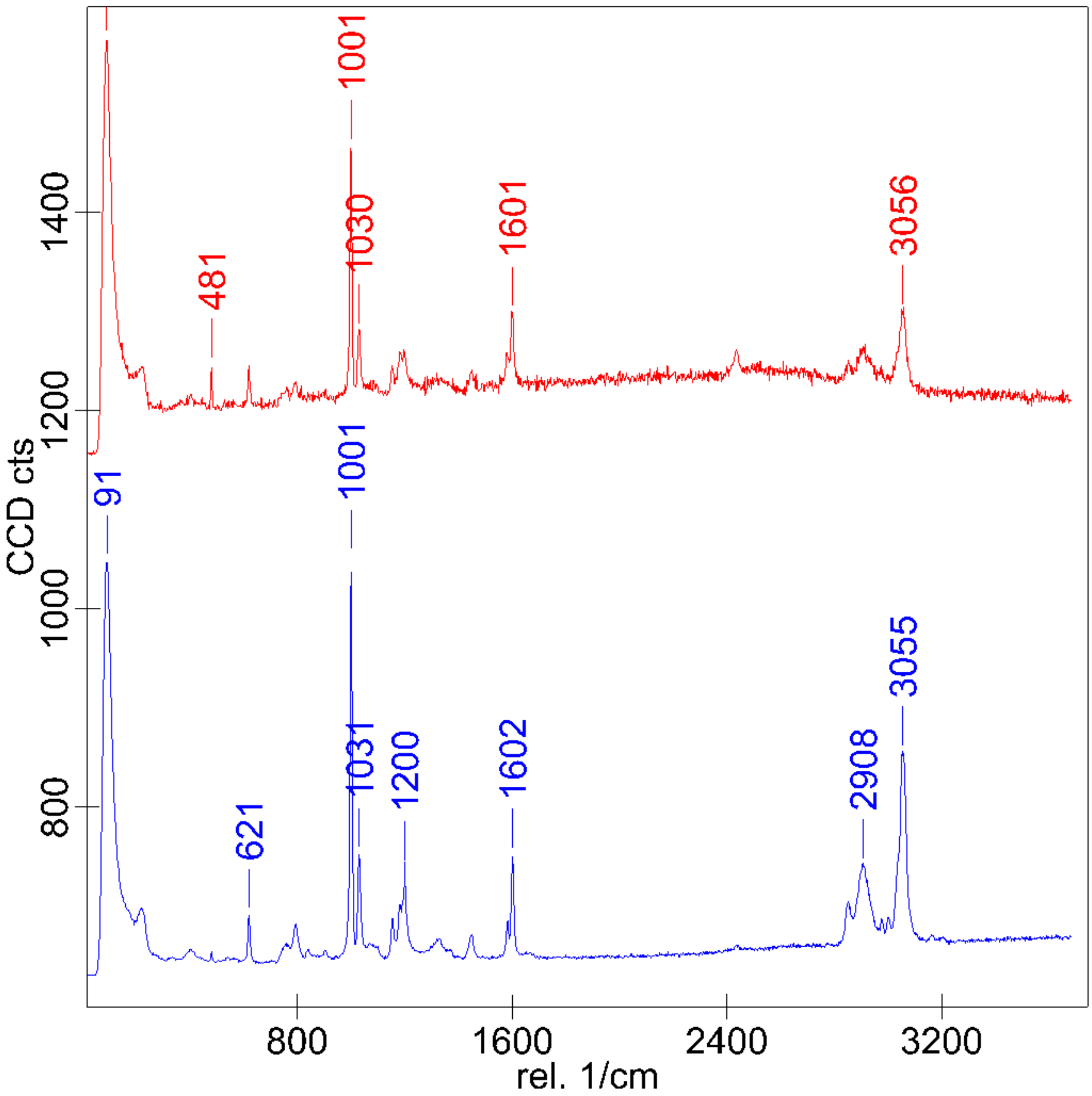

Figure 9: Exemplary spectrum of PS starting material (blue) fragmented PS (red).

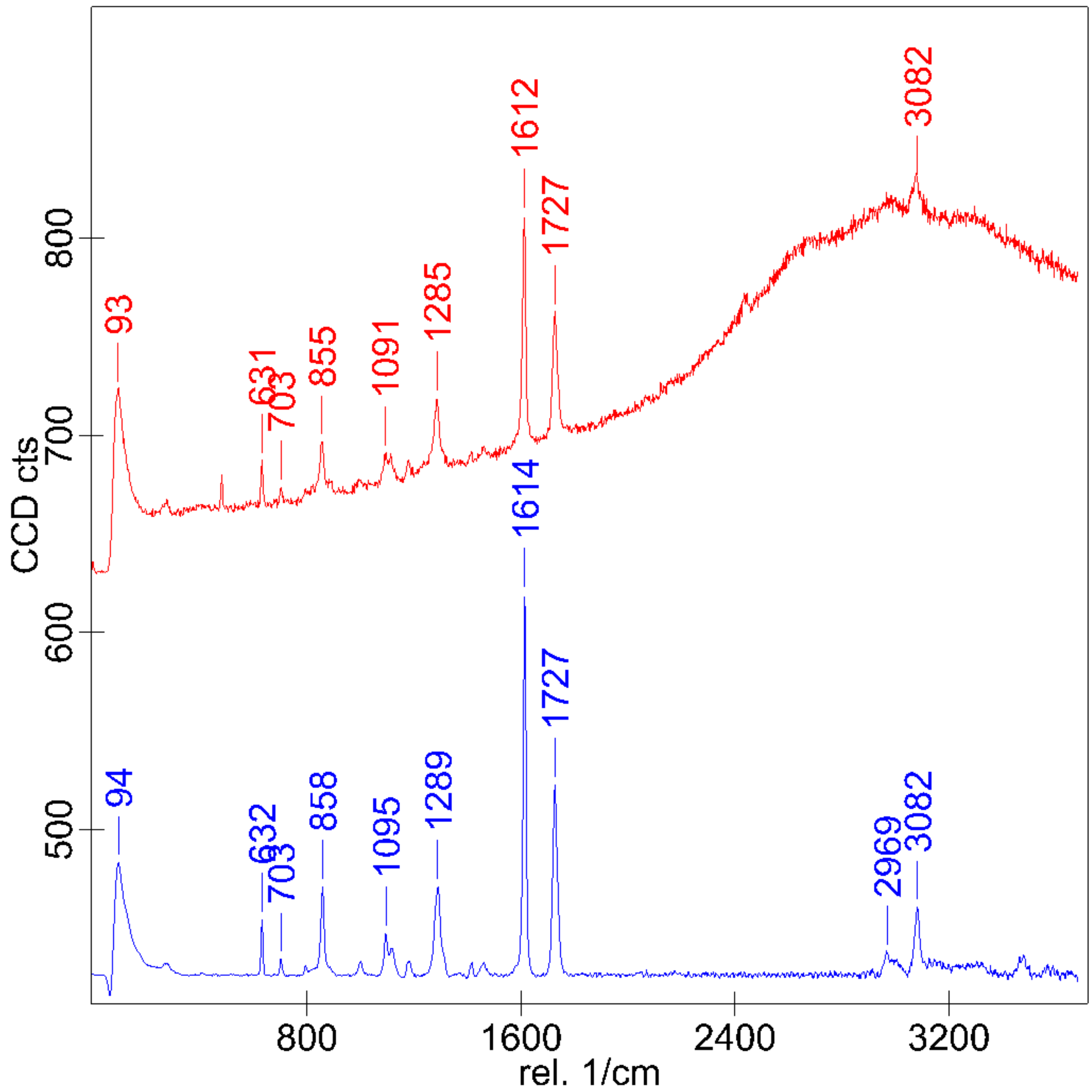

Figure 10: Exemplary spectrum of PET starting material (blue) fragmented PET (red).

Additional in-depth manual fiber analysis:

To confirm that the fibers detected in the automated analysis originate from the fragmentation of the original polymer piece additional manual measurements were performed. (Original automated analysis Jan. 2019, second manual analysis Sept 2019) We can confirm that PET, PLA and PS produce fragments in the shape of fibers. These fibers are typically shorter than 100 µm (for PET there was one exception see Figure 11) and could be identified through Raman microspectroscopy (Figure 11-14). We also found fibers from aerial contamination. These showed only a fluorescence signal and were typically larger than 100 µm. Examples for the recorded spectra and images of the fibers can be viewed in Figure 12. Since the samples could be remeasured after a 9-month storage (on gold coated polycarbonate filters in glass Petri dishes) we further conclude that the particles generated through sonication are stable for at least 9 moths

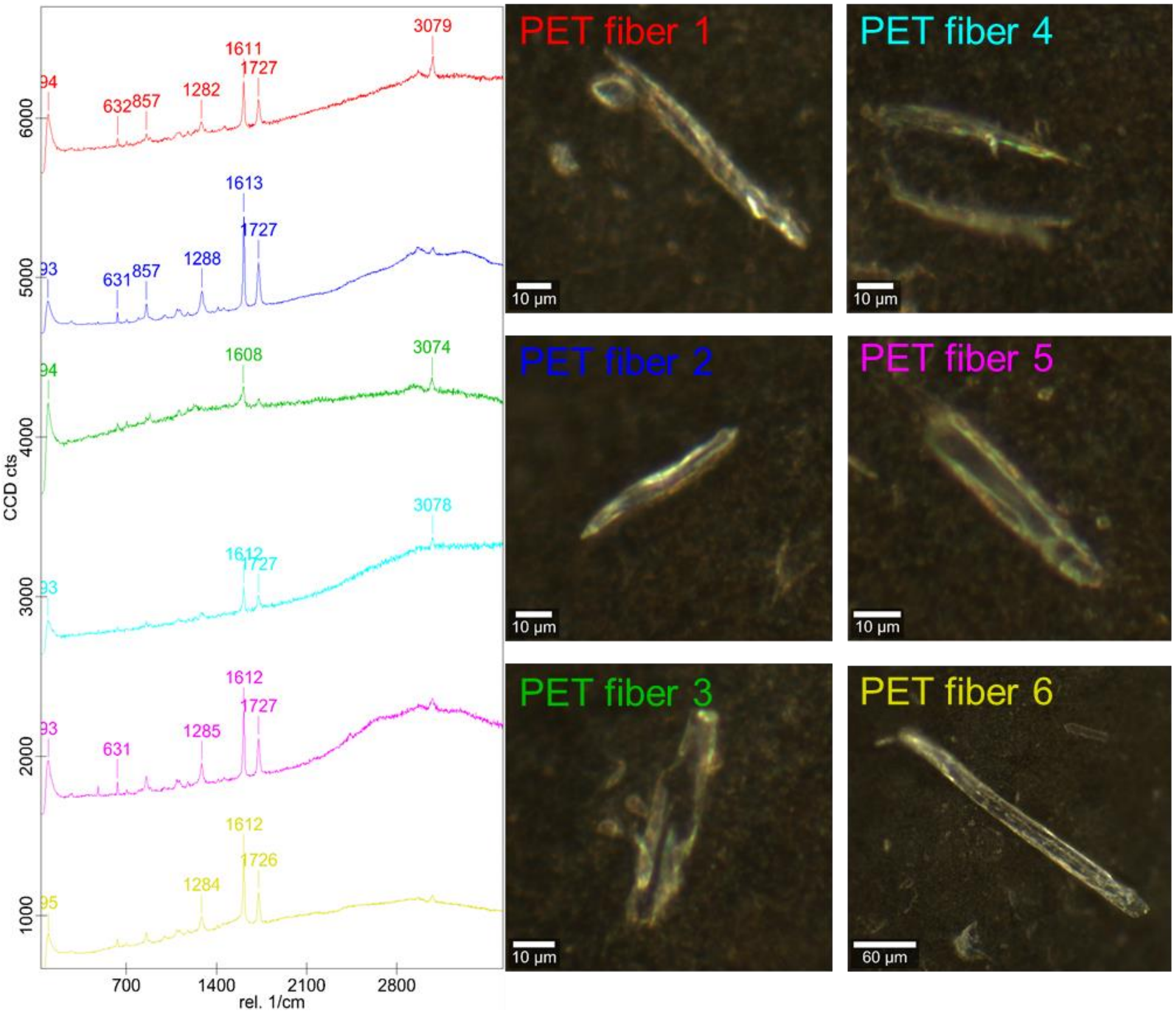

Figure 11: Fiber-like structures produced through sonication of PET.

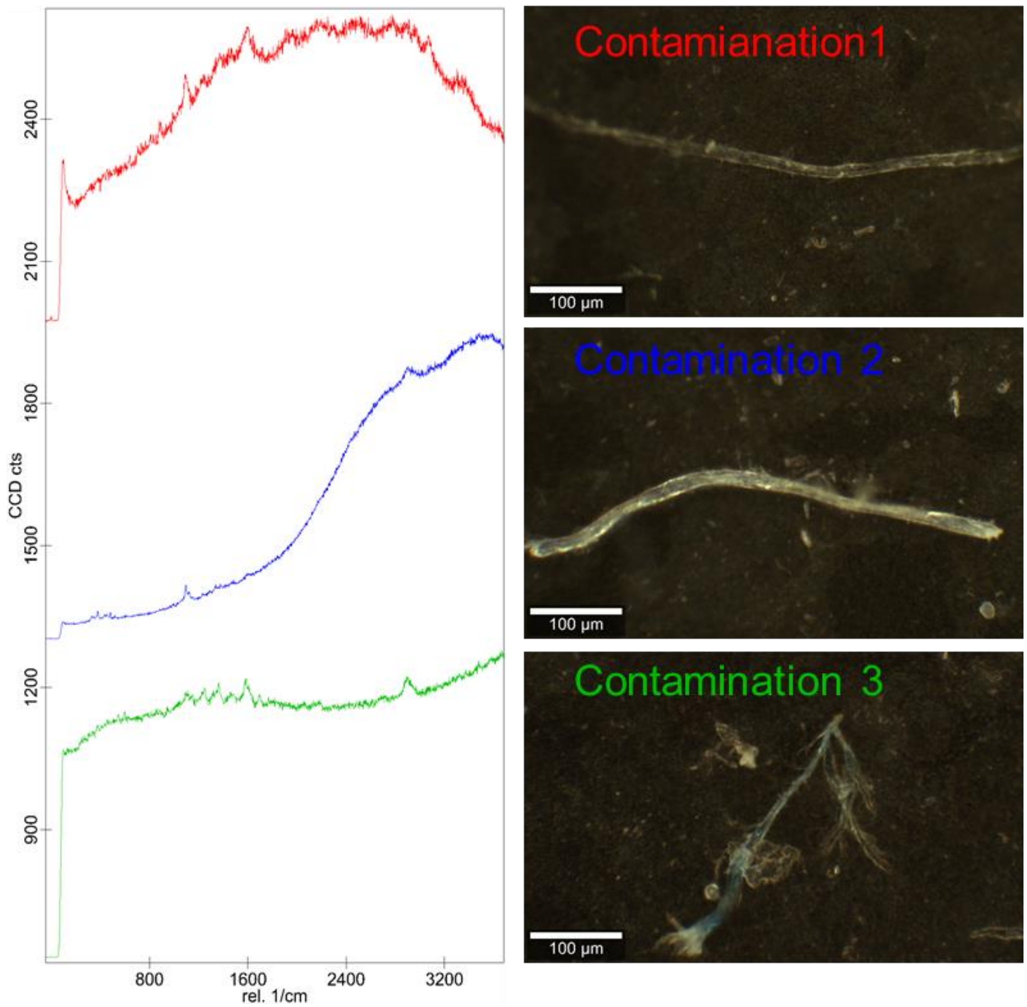

Figure 12: Fibers from aerial contamination.

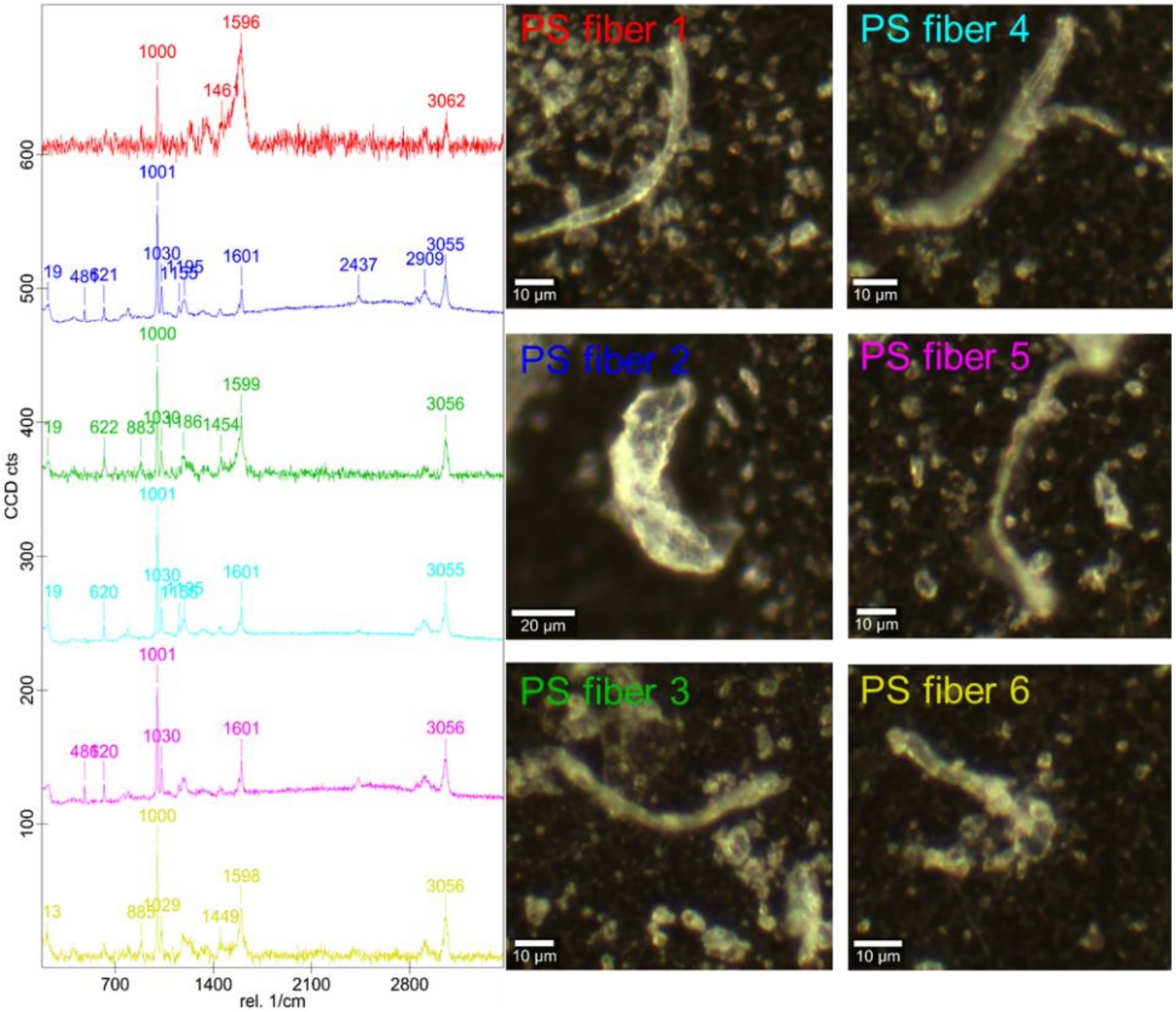

Figure 13: Fiber-like structures produced through sonication of PS.

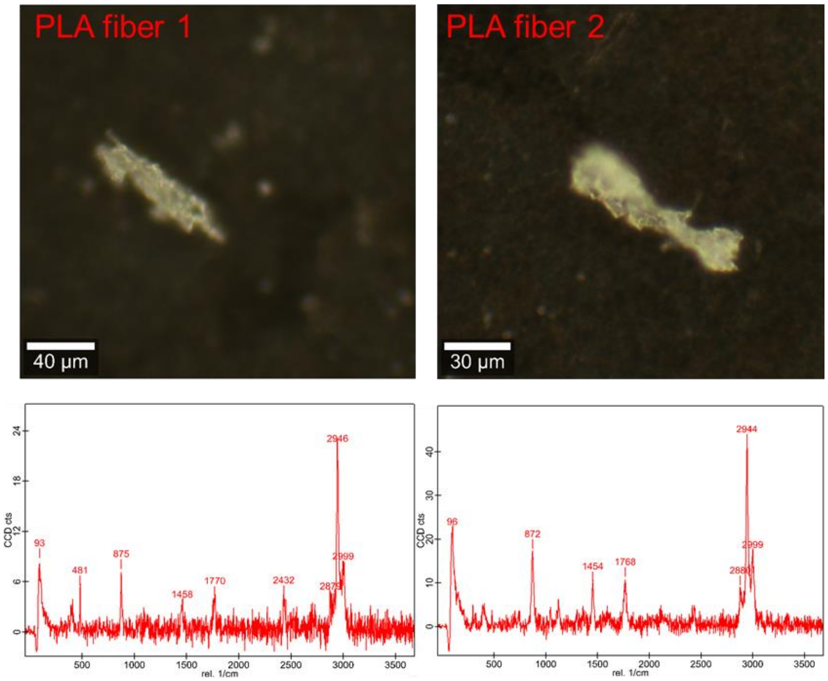

Figure 14: Fiber-like structures produced through sonication of PLA.

Size Distribution Statistics

Table 1: Size distribution statistics.

	Absolute Numbers			size distribution in %				descriptive statistics	
PS	Total	Particles	Fibers	>100	100-50	50-20	<20	Average size in µm	MAD in µm
Replicate 1	217328	214382	2946	0.36	2.37	19.33	77.94	16.47	8.45
Replicate 2	9227	8638	589	3.55	9.94	41.29	45.22	34.77	23.13
Replicate 3	61464	56613	4851	6.46	14.10	38.49	40.96	39.44	27.46
Mean R 1-3	96006	93211	2795	3.46	8.80	33.04	54.70	30.23	
SD absolute	108265	107644	2135	3.05	5.95	11.95	20.23	12.14	
SD percent	113	115	76						
PLA	Absolute Numbers			size distribution in %				descriptive statistics	
	Total	Particles	Fibers	>100	100-50	50-20	<20	Average size in µm	MAD in µm
Replicate 1	21067	20454	613	3.40	7.24	27.94	61.41	26.10	18.58
Replicate 2	10602	9924	678	6.92	12.76	36.95	43.37	39.03	28.07
Replicate 3	20757	19471	1286	3.16	10.06	34.96	51.82	30.99	20.17
Mean R 1-3	17475	16616	859	4.50	10.02	33.28	52.20	32.04	
SD absolute	5954	5817	371	2.11	2.76	4.73	9.03	6.53	
SD percent	34	35	43						
PET	Absolute Numbers			size distribution in %				descriptive statistics	
	Total	Particles	Fibers	>100	100-50	50-20	<20	Average size in µm	MAD in µm

Replicate 1	48882	47480	1402	0.70	4.76	28.89	65.65	20.97	11.62
Replicate 2	72075	68436	3639	3.53	10.30	36.76	49.41	31.34	19.85
Replicate 3	17586	16753	833	2.00	8.73	34.66	54.61	27.38	16.04
Mean R 1-3	46181	44223	1958	2.08	7.93	33.44	56.55	26.56	
SD absolute	27345	25995	1483	1.42	2.86	4.07	8.29	5.23	
SD percent	59	59	76						

Mechanistic Implications

Table 2: Preliminary data for PE, PP and PA for PVC see SI, Figure 5.

Polymer	Absolute Numbers			size distribution in %			
	Total	Particles	Fibers	>100	100-50	50-20	<20
PE	11795	11090	705	1.27	6.20	39.04	53.49
PP	83635	81617	2018	0.43	3.63	22.70	73.24
PA	could not be fragmented in alkaline solution and resulted in the formation of a gel						

Ultrasonic bath testing procedure

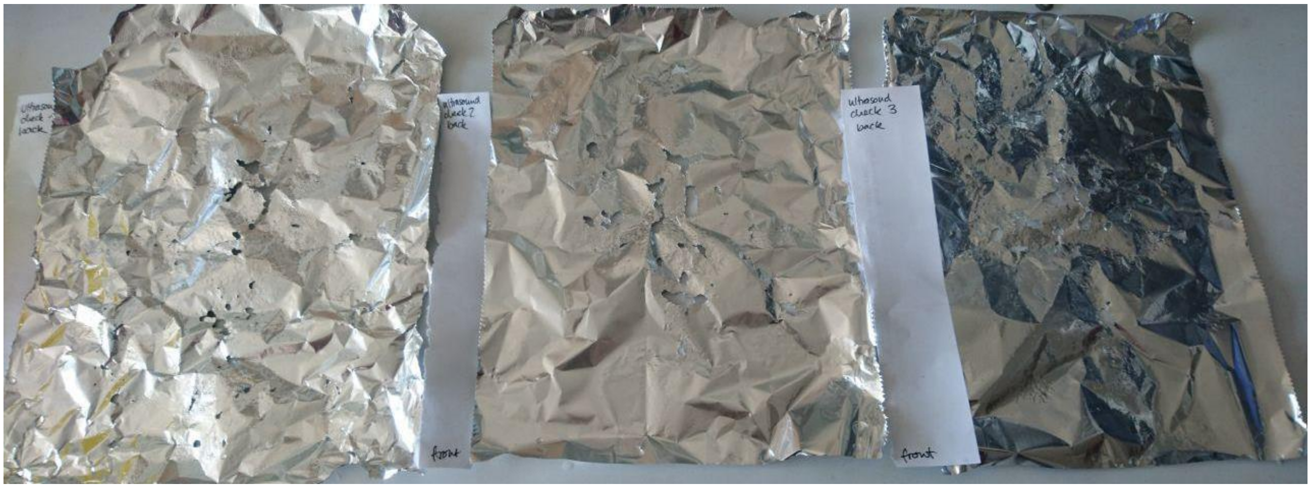

Figure 15: Identification of hotspots in ultrasonic bath with aluminum foil. Submerge the foil in the ultrasound bath and turn it on after a few seconds holes should appear in the surface. Leave the foil in for approximately one minute. The largest holes will indicate the strongest field. After finding these hotspots all samples should be placed in exactly this position to ensure identical fragmentation conditions.

Axial resolution of the Measurements

ATR and Reflectance IR Spectroscopy

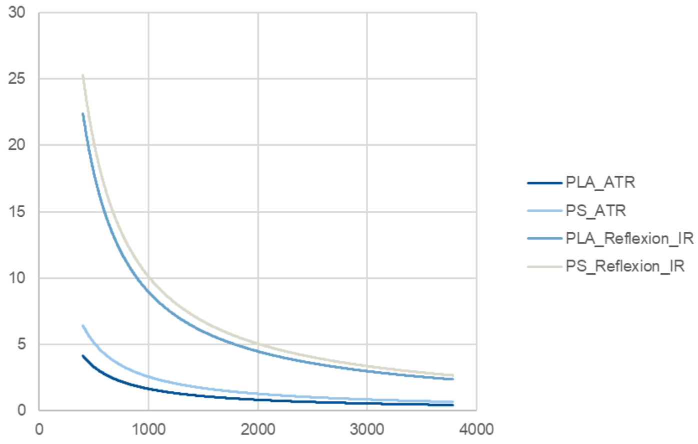

pi	3.14159265
n(ATR)	2.4
sin2(45)	0.5
n(Sample)	1.4 PLA, 1.58 PS 1.57 PET
n(ratio)	0.58333333

$$d_p = \frac{\lambda}{2\pi n_1 \sqrt{\sin^2(\Theta) - (n_2/n_1)^2}}$$

Raman spectroscopy with a 532 Laser

Sample and instrumental properties			Sample	λ in cm	axial resolution in cm	axial resolution in µm
n(PS)	1.58	refractive	PS	5.32E-05	2.10E-03	2.1014
n(PET)	1.57	index in	PET	5.32E-05	2.09E-03	2.0881
n(PLA)	1.4	g/cm	PLA	5.32E-05	1.86E-03	1.862
N.A.	0.4	numerical aperture				

$$\Delta = \frac{4 \cdot n \cdot \lambda}{(N.A.)^2}$$

Figure 16: In order to perform a sucsessful analysis and to interpret the results it is imortant to know what exactly you are measuring and at what depth. We have put togerther our penetration depths for ATR-FTIR, Reflectance FTIR and Raman microspectroscopy with a 532 nm laser.

μ-FTIR analysis

PET analysis

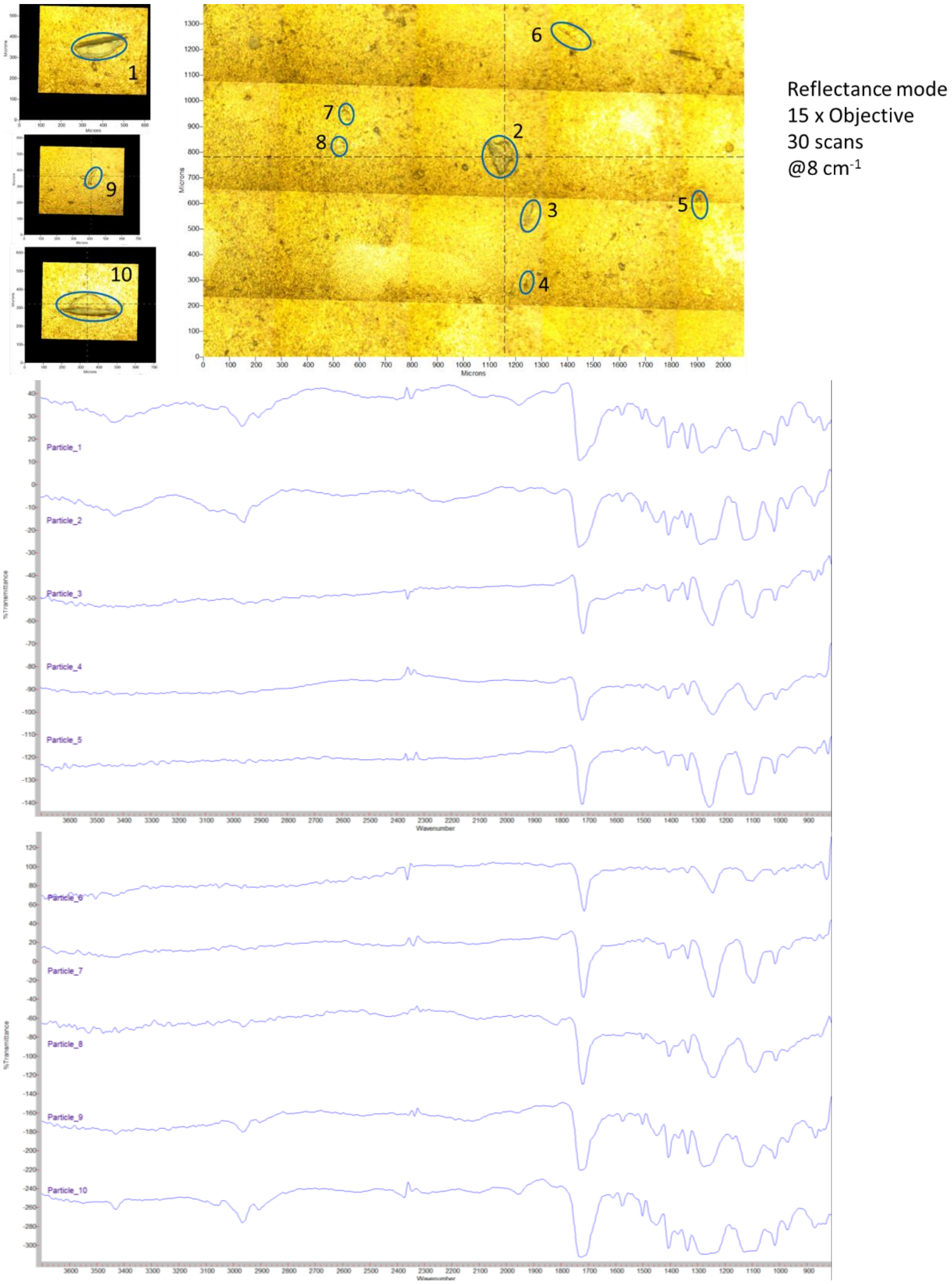

Figure 17: Image of the analyzed particles (top). The particle numbers correspond to the spectra below. All measurements were conducted on a μ-FTIR system by Agilent Cary 620.

PLA analysis

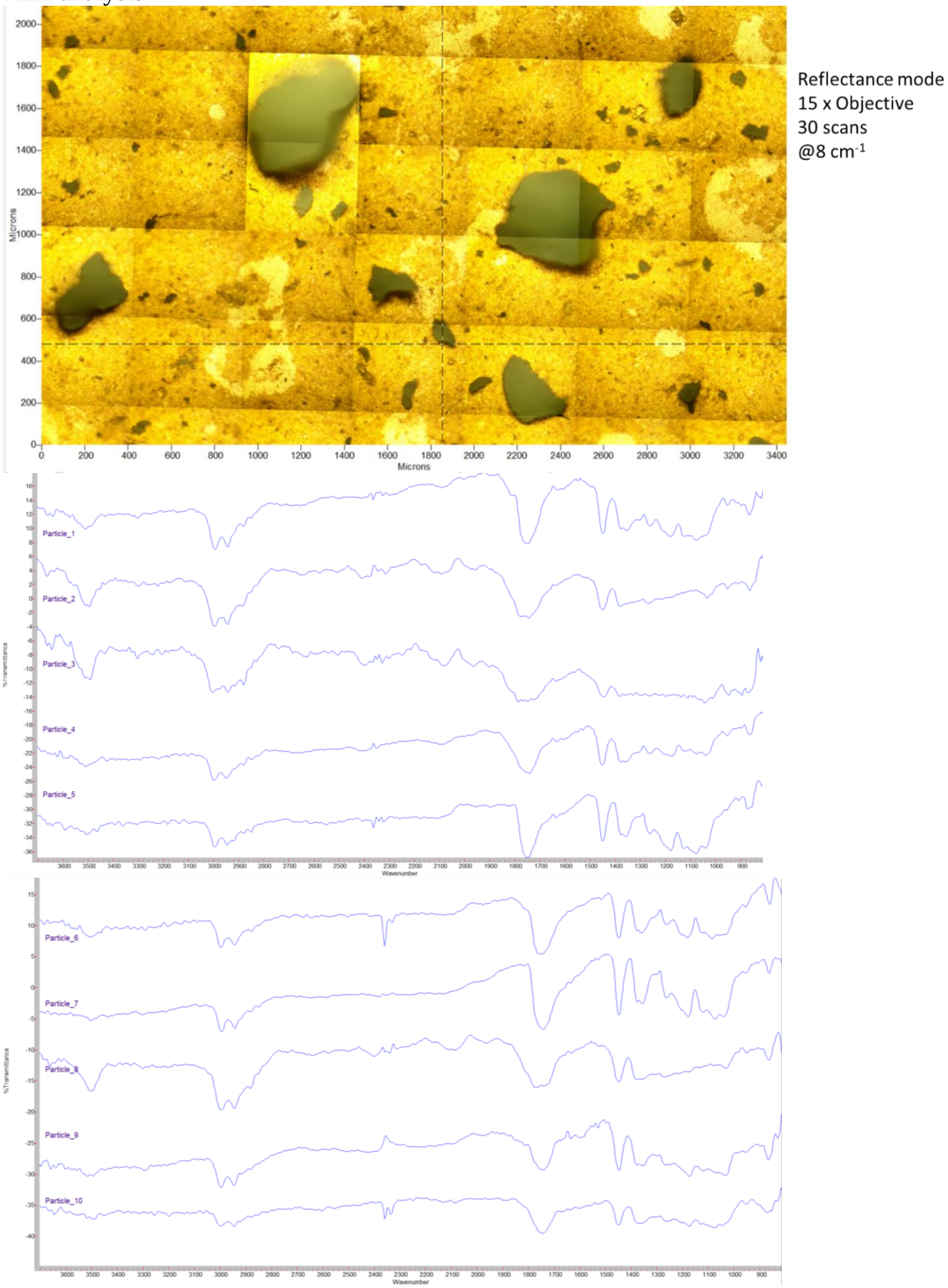

Figure 18: Image of the analyzed particles (top). The particle numbers correspond to the spectra below. All measurements were conducted on a µ-FTIR system by Agilent Cary 620.

PS analysis

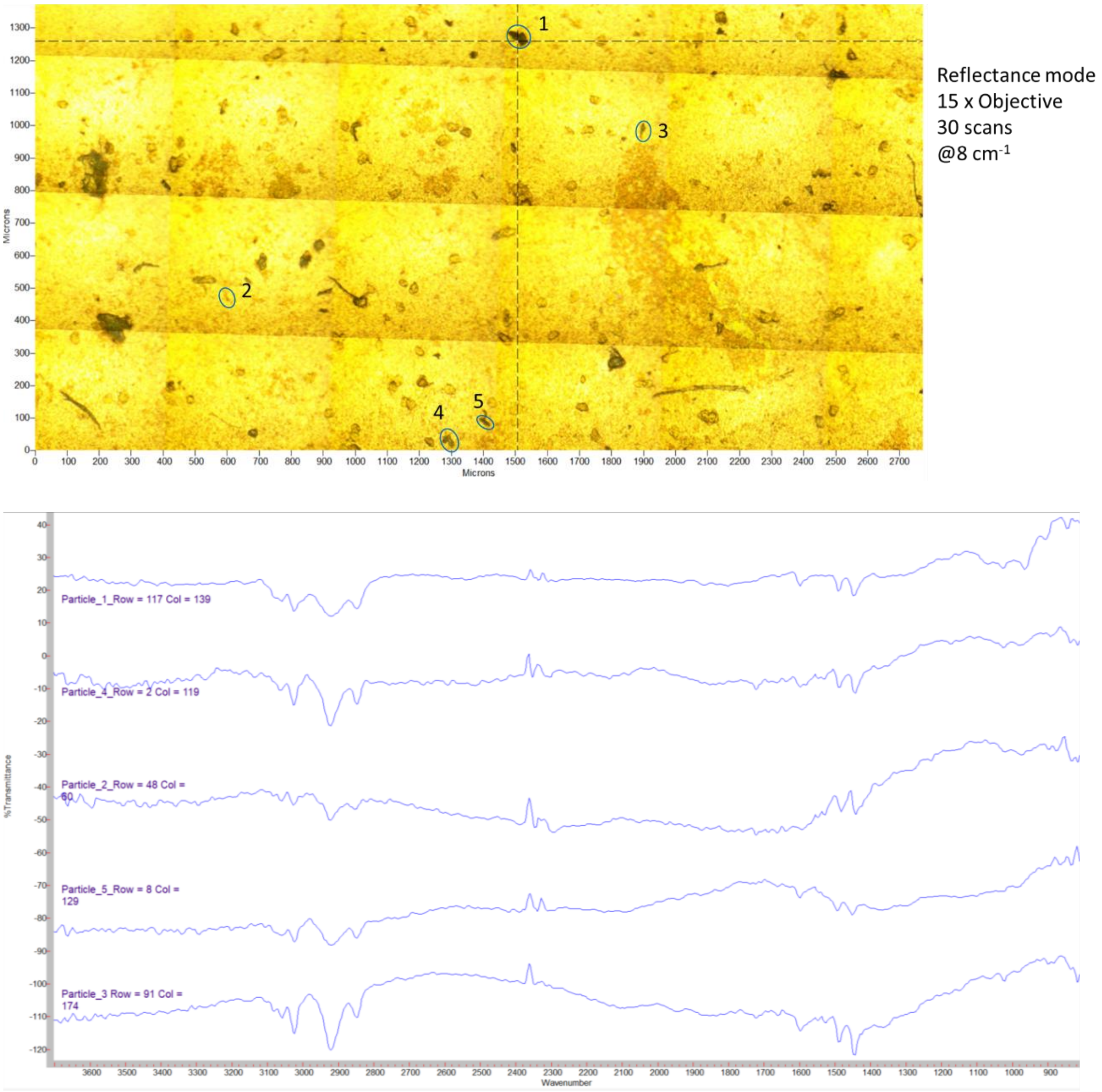

Figure 19: Image of the analyzed particles (top). The particle numbers correspond to the spectra below. All measurements were conducted on a μ-FTIR system by Agilent Cary 620.

[1] Elisabeth von der Esch, Alexander J. Kohles, Philipp M. Anger, Roland Hoppe, Reinhard Niessner, Martin Elsner, and N.P. Ivleva, TUM-ParticleTyper: A Detection and Quantification Tool for Automated Analysis of (Microplastic) Particles and Fibers. PLOS ONE submitted
